# Hyperthermia, Cytotoxicity, and Cellular Uptake Properties of Manganese and Zinc Ferrite Magnetic Nanoparticles Synthesized by a Polyol-Mediated Process

**DOI:** 10.3390/nano9101489

**Published:** 2019-10-18

**Authors:** Cristian Iacovita, Adrian Florea, Lavinia Scorus, Emoke Pall, Roxana Dudric, Alin Iulian Moldovan, Rares Stiufiuc, Romulus Tetean, Constantin Mihai Lucaciu

**Affiliations:** 1 Department of Pharmaceutical Physics-Biophysics, Faculty of Pharmacy, “Iuliu Hatieganu” University of Medicine and Pharmacy, Pasteur 6, 400349 Cluj-Napoca, Romania; cristian.iacovita@umfcluj.ro (C.I.); scorus.lavinia@gmail.com (L.S.); rares.stiufiuc@umfcluj.ro (R.S.); 2 Department of Cell and Molecular Biology, Faculty of Medicine, “Iuliu Hațieganu” University of Medicine and Pharmacy, Pasteur 6, 400349 Cluj-Napoca, Romania; aflorea@umfcluj.ro; 3 Department of Reproduction Obstetrics and Veterinary Gynecology, University of Agricultural Sciences and Veterinary Medicine, Manastur 3-5, 400372 Cluj-Napoca, Romania; pallemoke@gmail.com; 4 Faculty of Physics, “Babes Bolyai” University, Kogalniceanu 1, 400084 Cluj-Napoca, Romania; roxana.pacurariu@phys.ubbcluj.ro (R.D.); romulus.tetean@phys.ubbcluj.ro (R.T.); 5 Department of Bionanoscopy, MedFuture Research Center for Advanced Medicine, “Iuliu Hatieganu” University of Medicine and Pharmacy, Pasteur 4-6, 400337 Cluj-Napoca, Romania; alin.moldovan@umfcluj.ro

**Keywords:** manganese and zinc ferrite magnetic nanoparticles, ethylene glycol, polyol method, magnetic hyperthermia, specific absorption rate, cancer cell uptake, endocytosis, cytotoxicity, biodegradation

## Abstract

Manganese and zinc ferrite magnetic nanoparticles (MNPs) were successfully synthesized using the polyol method in ethylene glycol and were found to have high saturation magnetization values (90–95 emu/g at 4 K) when formed by ~30-nm crystallites assembled in an ~80-nm multicore structure. Hyperthermia data revealed a sigmoidal dependence of the specific absorption rate (SAR) on the alternating magnetic field (AMF) amplitude, with remarkable saturation SAR values in water of ~1200 W/g_Fe+Mn_ and ~800 W/g_Fe+Zn_ for the Mn and Zn ferrites, respectively. The immobilization of the MNPs in a solid matrix reduced the maximum SAR values by ~300 W/g_Fe+Mn, Zn_ for both ferrites. The alignment of the MNPs in a uniform static magnetic field, before their immobilization in a solid matrix, significantly increased their heating performance. Toxicity assays performed in four cell lines revealed a lower toxicity for the Mn ferrites, while in the case of the Zn ferrites, only ~50% of cells were viable upon their incubation for 24 h with 0.2 mg/mL of MNPs. Cellular uptake experiments revealed that both MNPs entered the cells in a time-dependent manner, as they were found initially in endosomes and later in the cytosol. All of the studied cell lines were more sensitive to the ZnFe_2_O_4_ MNPs.

## 1. Introduction

The last decades have shown an exponentially increased interest in the applications of magnetic nanoparticles (MNPs) in various fields [1]. Biomedical applications of magnetic nanoparticles include targeted drug delivery, magnetic hyperthermia (MH), contrast agents for magnetic resonance imaging (MRI), biological separation, neural stimulation, biosensing, and gene transcription [2]. On the basis of the fact that iron oxide MNPs, mainly magnetite (Fe_3_O_4_) and maghemite (γ-F_2_O_3_), possess high biocompatibility [3] since they are the only class of MNPs approved for clinical use by the US Food and Drug Administration [4], a huge interest has been devoted to improving methods for obtaining iron oxide MNPs with controlled sizes, size distributions, shapes, coating functionalizations, and magnetic properties. Commercial MNPs are available as contrast agents (e.g., Feridex, Resovist) and magnetic hyperthermia heating mediators (e.g., Nanotherm™). However, iron oxide MNPs have had limited value in magnetic moments (saturation magnetization) and relaxivity, resulting in limited heating capabilities and lower sensitivity in MRI diagnostics [5,6]. To overcome these limitations, new types of MNPs have been actively pursued, aimed at achieving improved magnetic properties such as magnetic anisotropy and saturation magnetization (*M_s_*), which are of paramount importance for increasing effectiveness, especially in MH applications [7].

The heating abilities of MNPs exposed to alternating magnetic fields in MH are quantified by the specific absorption rate (SAR) parameter, which is measured by the heat released in unit time by the unit mass of the MNP [8]. SAR values depend on both extrinsic and intrinsic parameters. The first category includes external AMF magnetic field parameters such as frequency (*f*), amplitude (*H*), and medium viscosity (*η*). While most researchers consider SAR values to increase linearly with AMF field frequency whatever the size and type of the MNPs, the SAR dependence on *H* is much more complicated. In the case of small (<10 nm) superparamagnetic nanoparticles, SAR values are proportional to *H^2^* [8], while for larger MNPs, other power laws have been found to describe the SAR dependence on *H* [9]. For particles in the ferromagnetic regime, at high magnetic field strengths (*H* > 30–40 kA/m), the SAR values saturate [10]. These results show clearly that the heat released in MH cannot increase indefinitely up to the desired therapeutic values, just by controlling the external field parameters. Moreover, the Brezovich criterion [11] sets a safety limit on the frequency and amplitude of the AC magnetic field for human exposure by limiting the product between these two parameters to 5 × 10^8^ Am^−1^s^−1^ due to the fact that both high-frequency and high-amplitude AMFs produce eddy currents in conducting media and can lead to nonspecific heating and/or damage to the human body. Other authors have proposed a limit one order of magnitude higher, 5 × 10^9^ Am^−1^s^−1^, considering that smaller areas treated or shorter field exposure must be better tolerated by patients [12].

The MNP intrinsic parameters able to influence the SAR values are size, size distribution, shape, and composition (including the surface coating), which finally dictates their magnetic properties and their behavior in an AMF magnetic field. The main magnetic parameters characterizing MNPs are *M_s_* and magnetic anisotropy (*K*). *M_s_* depends on MNP size and composition, and overall anisotropy is driven by several contributions: crystal or magneto-crystalline anisotropy, shape anisotropy, and surface effects. Crystal anisotropy is an intrinsic parameter depending on the structure and composition of MNPs and is primarily due to spin–orbit coupling. Three different strategies have been identified in the literature that have successfully elaborated on biocompatible MNPs with increasing magnetic properties and better MH performance.

### 1.1. Doping Iron Oxides with Other Transition Metals

Iron oxide MNPs exhibit a spinel structure, AB_2_O_4_. The cations reside either in the tetrahedral (A) or octahedral (B) sites of an FCC lattice formed by O_2_. The spinel could be either normal (M^2+^)[M^3+^]_2_O_4_, where the divalent cations are situated in the (A) sites, or inverse (M^3+^)[M^2+^M^3+^]O_4_, where the divalent cations occupy half of the (B) sites. In the case of the most known iron oxide, magnetite, the structure is an inverse spinel one, and Fe^2+^ and Fe^3+^ are divalent and trivalent ions, respectively. Due to the double exchange between Fe^3+^ and Fe^2+^ ions, spins in (B) sites are aligned ferromagnetically, but they are aligned antiferromagnetically with Fe^3+^ in (A) sites. As a consequence, the net magnetic moment of this structure may be calculated as the difference between the moment in (B) and (A) sites (µ_oct_ − µ_tet_), and, in the case of magnetite, this yields 4µ_B_. The substitution of Fe^2+^ ions with Mn^2+^ ions (with the electronic configuration 3d^5^) increases the overall magnetic moment from 4µ_B_ to 5µ_B_ [13,14,15,16,17,18].

Zinc ferrite is a normal spinel, with Zn^2+^ ions being in (A) sites. Therefore, pure zinc ferrite is an antiferromagnetic compound. Referring now to the magnetite structure, if (A) tetrahedral sites are doped with zinc (up to 30–40%), the overall *M_s_* increases. Replacing the magnetic iron ions in the tetrahedral Zn^2+^ reduces the µ_tet_, while the Fe^3+^ ions migrate to (B) sites, increasing in this manner the µ_oct_ [19,20,21,22].

### 1.2. Synthesizing Faceted MNP 

Iron oxide MNPs exhibit multiple facets featuring many edges and corners. This type of curved morphology displays many disordered surface spins due to large surface canting effects and consequently high surface anisotropy, which affects the heat dissipation properties of spherical MNPs [19]. Therefore, a second strategy was directed toward tuning the effective anisotropy of MNPs by modifying their shape or aspect ratio [23,24,25]. In this regard, iron oxide MNPs of different shapes (cubes [26,27,28], octopods [29], octahedrons [30], rods [31,32], discs [33], rings [34], and polyhedrons [35]) have been synthesized, and they exhibited better hyperthermia performance than their spherical counterparts.

### 1.3. Tuning the Size and the Organization of MNPs 

For small superparamagnetic MNPs, the heating efficiency in AC magnetic fields is related to the Neel and Brown relaxation. The SAR values for superparamagnetic nanoparticles are in the order of a few hundred W/g. Increasing the size of MNPs drives them from a superparamagnetic to a ferromagnetic state with SAR values almost one order of magnitude higher than the superparamagnetic ones. Their MH properties are dictated by their dynamic hysteresis behavior [10], which, besides the two relaxation mechanisms, is driven also by their DC magnetic hysteresis. Thus, through the optimization of hysteresis losses by fine-tuning the size of MNPs, their heating performance might well be improved [9,36,37]. The stabilization of MNPs may occur as single entities or as multicore aggregates. Flower-shaped MNPs with coherent crystallographic orientations have been reported with improved SAR values [38,39], probably due to collective spin rotation [40].

In this paper, we report on the synthesis of Mn and Zn ferrite MNPs using a polyol-based method. Among the various synthetic methods that have been explored so far for the synthesis of MNPs, the polyol one offers important advantages, as it allows for producing high-crystalline hydrophilic MNPs in one step and in a cost-efficient manner that can be easily scaled up [27,35]. It has also been demonstrated that the polyol process can provide control over the morphology of MNPs, ranging from single-core spherical MNPs to more complex multicore nanostructures, including hollow spheres and nanoflowers [18,39,41,42]. Nevertheless, the SAR values of MNPs are the result of several heating mechanisms. We performed hyperthermia measurements for both types of ferrites in water as a colloidal suspension and as a solid dispersion in polyethylene glycol 8000 (PEG 8K) (freezes below 60 °C), aiming at evaluating the contribution of the Brown relaxation mechanism to the MNPs’ overall heating efficiency.

We also took advantage of this particular setup, in which the MNPs were initially uniformly dispersed in liquid PEG 8K at 80 °C, and after that, the suspensions were frozen and cooled down to room temperature to study the effect of the MNP concentration and their collective behavior on MH. In this sense, measurements were performed on both randomly dispersed MNPs or MNPs aligned in an external DC magnetic field before and after freezing of the sample.

Another important issue addressed in this study is the cytotoxicity of Mn and Zn ferrites compared to their pure iron oxide counterparts that are synthesized and coated in the same conditions [35], as it is largely thought that iron oxide MNPs possess a much higher biocompatibility compared to other MNPs. The interactions of Mn and Zn ferrite MNPs with four different cell lines (human retinal pigment epithelial cells (D407), human lung carcinoma cells (A549), human melanoma cells (MW35), and mouse melanoma cells (B16F10)) were considered. The uptake of Mn and Zn ferrite MNPs incubated with the cells for up to 24 h by the four cell lines was monitored by using transmission electron microscopy (TEM) and was compared to the uptake of magnetite MNPs synthesized in the same manner in ethylene glycol. MNP cytotoxicity was assessed using a standard 3-(4,5-dimethylthiazol-2-yl)-2,5-diphenyltetrazolium bromide (MTT) assay up to a concentration of 0.2 mg/mL.

## 2. Materials and Methods

### 2.1. Synthesis Method

All the reagents employed in this study were of analytical grade and were used without any further purification. The magnetic nanoparticles were synthesized by using the following products: iron(III) chloride hexahydrate (FeCl_3_ 6H_2_O) (≥98%), manganese(II) chloride monohydrate (MnCl_2_ H_2_O) (≥98%), zinc(II) chloride (ZnCl_2_) (≥98%), ethylene glycol (EG) (≥99%), and sodium acetate trihydrate (NaAc) (≥99.5%) all reagents being purchased from Roth, Karlsruhe, Germany.

A conventional polyol-mediated synthetic route was applied for the preparation of ferrite-based MNPs as follows: 0.34 g of FeCl_3_ 6H_2_O, 0.1 g of MnCl_2_ H_2_O (ZnCl_2_), and 3.6 g of NaAc were mixed and dissolved in 40 mL EG. After the solutions were stirred thoroughly at room temperature for about 30 min, they were transferred in glass beakers (fitted in a stainless steel autoclave) that were sealed and introduced into a programmable controlled oven. The samples were heated at a rate of 3 °C/min up to 200 °C and were maintained at this temperature for 6 h. Finally, after the samples were allowed to cool down at room temperature, the black precipitates obtained were washed with double-distilled water at least 3 times and redispersed in water for further analysis. To improve the colloidal stability of the MNPs and hamper the formation of big clusters, 4 mg of each powder of MNPs was dissolved in 1 mL aqueous solution that was 25% tetramethylammonium hydroxide (TMAOH), sonicated for 1 h, and left overnight. Subsequently, the MNPs were magnetically separated and redispersed in double-distilled water. This operation was repeated twice. Upon this protocol, the TMAOH molecules attached to the MNP surface through the OH groups, thus preventing their further agglomeration into a big cluster and their sedimentation at the bottom of the vial.

### 2.2. Methods

TEM images of MNPs were acquired on a Hitachi HT7700 (Hitachi Ltd. Tokyo, Japan) microscope equipped with an 8-megapixel CCD camera and operated at 100 kV in high-contrast mode. A few microliters of MNP suspension were deposited on carbon-coated copper grids, the excess water was removed by filter paper, and the sample was allowed to dry at room temperature. The MNP size and size distribution was measured by using ImageJ free software (U.S. National Institutes of Health, Bethesda, MD, USA). A size histogram and fitting with a log-normal distribution was made with Microcal Origin Pro 2016 Software (OriginLab Corporation, Northampton, MA, USA).

X-ray diffraction (XRD) measurements were performed on dried powder samples at room temperature on a Bruker D8 Advance diffractometer using Cu Kα radiation. FullProf software was used for calculating the lattice parameters and phase percentages.

Fourier-transform infrared (FTIR) spectra of MNPs and TMAOH were recorded with a TENSOR II instrument (Bruker Optics Inc., Billerica, MA, USA) in attenuated total reflectance mode using the platinum attenuated total reflectance (ATR) accessory with a single reflection diamond ATR in the 400–4000 cm^−1^ spectral range with a resolution of 4 cm^−1^. A few microliters of either the 25% TMAOH solution or the MNP colloids were allowed to dry on the diamond crystal, and an average spectrum of 16 scans was recorded for each sample. Zeta potential measurements were taken using a Zetasizer Nano ZS90 (Malvern Instruments, Worcestershire, UK) in a 90° configuration.

Magnetic measurements were assessed with a Cryogenic Limited (London, UK) vibrating sample magnetometer (VSM) used on powder samples in the 4–300 K temperature range.

Hyperthermia properties of the magnetic nanoparticles were measured calorimetrically. AC magnetic fields (field strengths between 5 and 65 kA/m at a frequency of 355 kHz) were generated by an 8-turn coil that was made of copper and water-cooled and that was connected to a magnetic heating system, the Easy Heat 0224 from Ambrell (Scottsville, NY, USA). The samples, which were usually 0.5 mL of MNPs suspended in water or dispersed in solid PEG 8K at different concentrations, were placed in the center of the coil and were thermally insulated. The temperature was measured using a fiber-optic probe introduced into the center of the sample. Details about the experimental setup, magnetic field calibration, and SAR calculation are provided in the Supplementary Materials and in Reference [27].

### 2.3. Cell Lines

MNP cytotoxicity and cellular uptake was tested on 1 normal cell line and 3 cancer cell lines: human retinal pigment epithelial (D407) cells, a human melanoma cell line (MW35), a mouse melanoma cell line (B16F10), and a human lung adenocarcinoma (A549) cell line. All cell lines were obtained from American Type Cell Collection (ATCC) (Manassas, VA, USA). Details about the cell cultures are provided in the Supplementary Materials.

### 2.4. Cytotoxicity Assays

Cell survival in the presence of MNPs was tested with an MTT (3-(4,5-dimethylthiazol-2-yl)-2,5-diphenyl tetrazolium bromide; Sigma-Aldrich) assay. In this sense, the cell lines were plated (1 × 10^5^ cells/well) in 96-well plates for 24 h in normal propagation media. The two types of nanoparticles (in concentrations of 0.2 mg/mL, 0.1 mg/mL, and 0.05 mg/mL) were added to the complete medium. The cells’ behavior was observed with an inverted optical microscope (Nikon TS100, Nikon Instruments, Wien, Austria). After 24 h of exposure to the MNPs, cell viability was assessed by adding 0.5 mg MTT to each well. The formazan particles formed over 1 h of incubation at 37 °C were dissolved with dimethyl sulfoxide (DMSO) (Sigma-Aldrich, St. Louis, MO, USA), and the absorbance was read at 550 nm using a microplate reader (Bio-Rad, Hercules, CA, USA). The survival percentages were calculated as the ratios to the values obtained for the untreated controls. All experiments were performed in triplicate. All data are reported as the mean value ± the standard error of the mean (SEM). A minimum of three replicates were performed for each experiment.

### 2.5. Cellular Uptake

The characteristics of the cellular uptake of MNPs were assessed by TEM image analysis after incubating all 4 cell lines with the highest concentration of MNPs (0.2 mg/mL) for 4 h and 24 h. The cells were processed for TEM as described in the Supplementary Materials and in Reference [35].

## 3. Results and Discussion

### 3.1. Structural Characterization

**Figure 1:**
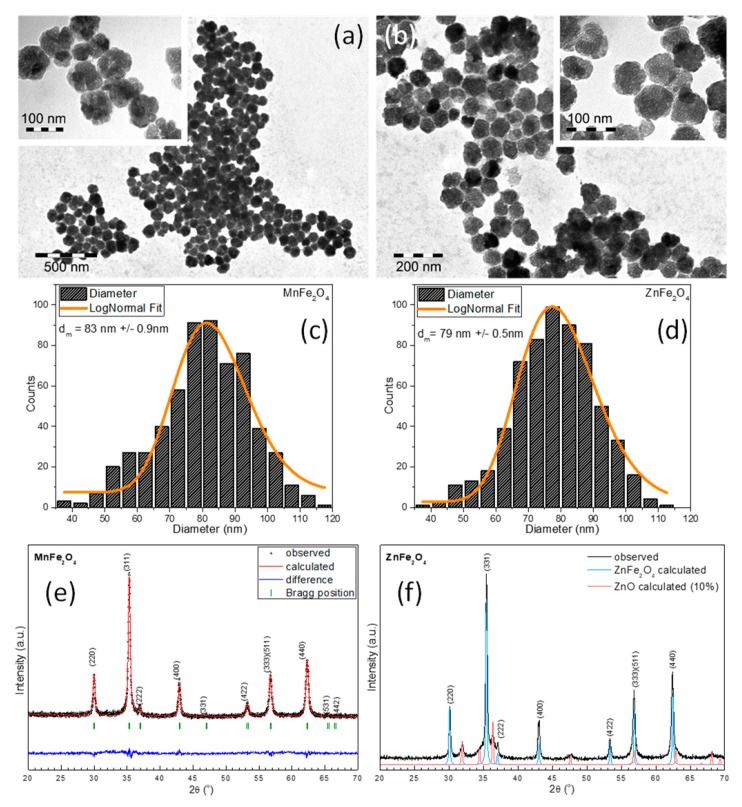
Large-scale TEM images of (**a**) MnFe_2_O_4_ magnetic nanoparticles (MNPs) and (**b**) ZnFe_2_O_4_ MNPs synthesized in ethylene glycol (EG). Insets are zoomed-in large-scale TEM images. Size distribution histograms of (**c**) MnFe_2_O_4_ MNPs and (**d**) ZnFe_2_O_4_ MNPs fitted to a log-normal distribution (orange lines) and their corresponding XRD diffraction patterns (**e**,**f**).

TEM images for both types of ferrite-based MNPs can be seen in Figure 1a,b. As one can observe, the MNPs presented irregular shapes. A small proportion of the MNPs was faceted. The frequency of the MNP sizes, determined by measuring the diameter of more than 500 MNPs based on TEM images (through ImageJ) and fitted using the log-normal distribution function, showed that the average diameter of spherical MNPs was approximately 83 nm and 79 nm for manganese- and zinc-based ferrite MNPs, respectively (Figure 1c,d). A closer inspection of the TEM images revealed that the individual spherical MNPs were composed of multicore MNPs (insets of Figure 1a,b). The existence of voids in the inner part of the spheres and the distribution of a multicore toward the edges of the spheres clearly indicated the formation of hollow spheres (insets of Figure 1a,b). As in the case of maghemite [43], the formation of this particular morphology of MNPs was attributed to the amount of 26 mmol of NaAc used in the reaction mixture.

To further analyze the crystalline structure of ferrite-based MNPs, X-ray diffraction (XRD) was performed on the powder samples. The XRD pattern revealed the existence of a cubic spinel crystalline structure in the case of manganese-based MNPs (Figure 1e). The position and relative intensities of all diffraction peaks were ascribed to MnFe2O4 ferrite (JCPDS No. 10-0319). No other peaks were found in the XRD pattern, indicating that all manganese-based MNPs were of pure MnFe_2_O_4_. Instead, the XRD pattern of zinc-based MNPs exhibited the coexistence of two phases (Figure 1f). The position and the relative intensities of the diffraction peaks were ascribed to the two phases of cubic spinel ZnFe_2_O_4_ (JCPDS No. 88-0315) and zinc oxide (JCPDS No. 89-0596). The corresponding lattice parameters, **a** = 8.429 Å and **a** = 8.431 Å for MnFe_2_O_4_ and ZnFe_2_O_4_, respectively, were very close to those of their bulk counterparts, **a** = 8.499 Å and **a** = 8.444 Å, respectively. In addition, these values were larger than the lattice parameter for bulk magnetite, which was **a** = 8.375 Å. The change in the lattice parameters was in agreement with the ionic radii of the doping ions. Both the Mn^2^+ and Zn^2+^ ions had larger radii compared to the other 3dn series (Mn^2+^ 0.080 nm, Zn^2+^ 0.074 nm, and Fe^3+^ 0.064nm) [44]. The noticeable increase in lattice parameter **a** was related to the higher tendency of the two doping ions to go to the A sites to accommodate these larger cations. The crystalline size of MnFe_2_O_4_ and ZnFe_2_O_4_, calculated as a mean from (220), (311), and (440) diffraction peaks using Scherrer’s formula, was 33.3 nm and 31.8 nm. As the sizes calculated from the XRD data corresponded with the smallest crystallites, the discrepancy between them and the mean diameters resulting from TEM clearly reflected the polycrystalline nature of these ferrite MNPs. Assuming a spherical shape of the crystallites and based on the relatively broad diameter distribution of the hollow spherical MNPs (between 65 and 95 nm), we can estimate that the multicore MNPs were assembled from 8–10 to 20–30 crystallites.

### 3.2. Magnetic Properties

**Figure 2:**
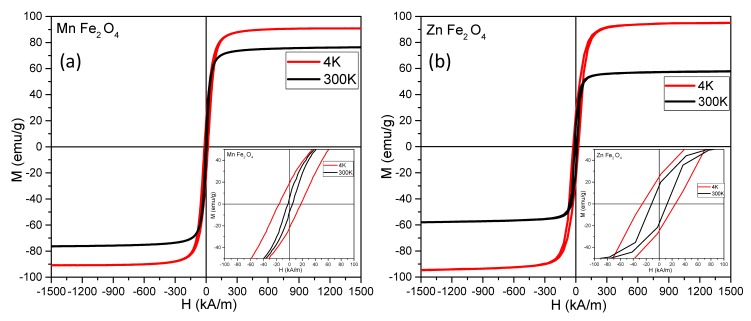
Magnetization curves (*M* = *f*(*H*)) for (**a**) MnFe_2_O_4_ MNPs and (**b**) ZnFe_2_O_4_ MNPs measured at 4 K and 300 K. Insets represent low-field regime hysteresis loops.

The magnetic response of MNPs was characterized both as a function of the field (M-H loops: Figure 2a,b) and as a function of the temperature (zero field cooled/field cooled ZFC/FC curves: Figure S1). The magnetic parameters (saturation magnetization (*M_s_*), coercive field (*H_c_*), and magnetic remanence (*M_r_*) extracted from the hysteresis cycles together with the calculated anisotropy constants are collected in Table 1.

At low temperature (4K), both types of MNPs showed hysteresis curves with similar shapes (Figure 2: red lines), revealing a standard ferrimagnetic character. Both the Mn and Zn ferrite MNPs synthesized in EG displayed an *M_s_* of 90 emu/g and 95 emu/g, which was significantly higher than the *M_s_* of Fe_3_O_4_ MNPs synthesized in EG (75 emu/g) [35]. It is important to note that the Fe_3_O_4_ MNPs synthesized in EG [35] in the same conditions as the Mn and Zn ferrite MNPs reported in this paper were also multicore MNPs but with TEM diameters of 270 nm, i.e., approximately three times larger compared to the Mn and Zn ferrites. Large MNPs are generally expected to display an *M_s_* close to the value obtained for the bulk material (for magnetite 92 emu/g), but in this case, the significantly lower *M_s_* might be attributed to the cation vacancies resulting from synthesis during the aggregation of multiple crystallites. However, both Mn and Zn ferrite MNPs presented high saturation magnetization values at 4 K, which were close to the bulk values. These observations are an indication, on one hand, of excellent crystallinity, and on the other hand, they are an indication that the cationic substitutions of Fe^2+^ with either Mn^2+^ or Zn^2+^ led to an increase in the magnetization of the MNPs, as expected.

The lowest coercive field at 4 K was obtained for the Mn ferrites, a result that was in agreement with the well-known “softness” of manganese ferrites, while the magnetite and the Zn ferrites had almost the same *H_c_* (26 kA/m). At 300 K, there was a normal decrease in both *M_s_* and *H_c_* for all samples. For the Mn ferrites, the *M_s_* value at 300 K was 76 emu/g, very close to the value of 75 emu/g previously reported for this type of MNPs prepared in EG [43]. The largest decrease in the *M_s_* was measured for the Zn ferrites, with a value of 58 emu/g at 300 K.

As mentioned above, the stable phase of ZnFe_2_O_4_ possesses a normal spinel structure in which diamagnetic Zn^2+^ and magnetic Fe^3+^ ions are located in the (A)- and (B)-sites, respectively. Due to the negative superexchange interaction between Fe^3+^ ions only at (B)-sites, ZnFe_2_O_4_ with a normal spinel structure behaves as an antiferromagnet below 10 K and is paramagnetic above this temperature. However, our ZnFe_2_O_4_ MNPs exhibited ferrimagnetic properties with a nonzero coercive field and high magnetization even at room temperature, as shown in Figure 2b. This result can be explained only by considering a different crystal structure from the normal spinel for the zinc ferrites. Unusual behavior for Zn ferrites was observed as early as 1950 [45], when both experimental results and theoretical calculations showed that the magnetic moments for ferrous ferrite and manganese ferrites were 4 µ_B_/formula unit and 5 µ_B_/formula unit, respectively, and that in the case of nonmagnetic Zn^2+^ ferrites, the partial substitution of Fe^2+^ with Zn^2+^ ions (40–60%) could lead to magnetic momentum as high as 5.8 µ_B_/formula unit for such Zn ferrites. The random distribution of Zn^2+^ and Fe^3+^ ions in the (A)-sites as well as the (B)-sites and the presence of a strong superexchange interaction between them (giving rise to high magnetization at low temperatures and also at room temperature) were also observed for sputtered ZnFe_2_O_4_ films [46]. According to our XRD data, in the case of Zn ferrites, some of the Zn^2+^ ions were found in a ZnO phase, meaning that the Zn ferrite was not stoichiometric, and this fact could also explain the high *M_s_* of these MNPs. As the temperature increased, the Zn^2+^ ions might have migrated in the (A) sites, thus reducing the saturation magnetization to 58 emu/g. Our results are in agreement with recent experiments proving that Zn_0.3_Fe_2.7_O_4_ MNPs approximately 40 nm in diameter have a saturation magnetization of 89 emu/g at 5 K, which drops to about 40 emu/g at 300 K [47].

The ratios of *Mr/M_s_* were smaller than 1:2 for all the ferrites both at low and high temperatures, thus indicating uniaxial anisotropy. Therefore, the anisotropy constants were calculated as *K_eff_* = *µ_0_H_c_M_s_*/0.96 [48], with the saturation magnetization and the coercive field values measured at 4 K. The effective anisotropy constant at low temperatures was very close to the bulk magnetite values (11–13 kJ/m^3^) for the ferrous ferrite, indicating the good quality of our MNPs. A slightly smaller value of the effective anisotropy constant was obtained for the Mn ferrites, a behavior that was consistent with the lower anisotropic constant in the case of bulk Mn ferrite. For the Zn ferrites, the effective anisotropy constant of 17 kJ/m^3^ was high compared to the value for magnetite, showing that the substitution of Fe^2+^ ions with Zn^2+^ ions led to an increase in the anisotropy, which was in agreement with values reported by other groups [47].

In Table 1, it can be observed that the values of *H_c_* and *M_r_* at room temperature for both types of MNPs were significantly smaller compared to the ones measured at 4 K. However, the behaviors of ferrite MNPs were not superparamagnetic at room temperature, as can be clearly seen in the zero-field-cooled/field-cooled (ZFC/FC) magnetization curves (Figure S1). In both cases, the ZFC and FC curves started to join around 300 K. In addition, the maximum of the ZFC curves was located at the same temperature and was broadened. This behavior suggests a gradual transition from the ferromagnetic to the superparamagnetic state, with the majority of the MNPs being in a blocked state at 300 K, the temperature range where the hyperthermia experiments were conducted. It should be noted that the *M*(*T*) values of the FC curves for the Mn and Zn ferrite MNPs were 37 emu/g and 34 emu/g, respectively, which suggests that both types of ferrite MNPs can exhibit similar interparticle interactions that can influence SAR values.

### 3.3. Hyperthermia Properties

As demonstrated above, at least some of the MNPs were in a ferromagnetic state at room temperature and possessed a nonzero magnetic moment. Therefore, they could develop strong attractive interparticle interactions that favored the formation of aggregates. The heating efficiency of MNPs, as will be presented in detail later, is strongly dependent on good dispersion. To ensure colloidal stability, the MNPs were treated with tetramethylammonium hydroxide (TMAOH) before the determination of hyperthermia properties [35]. The attachment of TMAOH to the MNPs’ surface was assessed by ATR-FTIR (Figure S2). As can be observed in Figure S2, the characteristic TMAOH vibrations at 949 cm^−1^ (υ_asym_ C–N) and 1487 cm^−1^ (δ_asym_ CH_3_) could be seen in the MNP ATR-FTIR spectra.

The heating capabilities of MNPs were tested in a magnetic field with variable amplitudes between 5 kA/m and 65 kA/m in steps of 5 kA/m operating at a frequency of 355 kHz, the highest value in our setup. The sonication of samples for 30 s before each determination assured a uniform distribution of MNPs in the sample volume. Upon this protocol, the TMAOH molecules attached to the MNP surface through OH groups, thus preventing their further agglomeration into big clusters and their sedimentation at the bottom of the vial. The zeta potential of the MNPs at pH 6.9 (which was reached after washing and resuspending them in double-distilled water) was in the range of 33–41 mV. The SAR values were calculated by measuring the initial slope of the temperature versus time curves and normalizing them for the amount of metal (Fe + Mn or Fe + Zn) in each sample, considering the heat capacity of the medium, as briefly described elsewhere [35] and in the Supplementary Materials.

Heat release in a macroscopic sample of magnetic material is due only to magnetic hysteresis. For MNPs, two other processes leading to heat generation (losses) have been described phenomenologically: Neel relaxation and Brown relaxation [12]. Due to the complexity of SAR dependence on different extrinsic and intrinsic parameters of a hyperthermia experiment, several theoretical approaches have been developed. For small particles in a superparamagnetic regime, linear response theory (LRT), which was originally proposed by Rosensweig [8], considers that the magnetization of MNPs depends linearly on the applied magnetic field, with the proportionality factor being complex susceptibility. LRT predicts that SAR values increase linearly with the square of the amplitude of the AMF field, without any saturation effect. Another important result of LRT is that the maximum SAR increases with *M_s_* and with the volume of the MNPs. Please note that *M_s_* also increases with the volume of the MNPs. However, as the size of the MNPs increases, they pass from a superparamagnetic state to a ferromagnetic state, where the assumptions of the LRT theory are not valid.

Another theoretical approach for calculating SAR values was proposed by Stoner and Wohlfarth [48]. They considered a simplified model with only two orientations possible for magnetization. Magnetization can be reversed only by magnetic fields higher than a critical value (*H_k_*). The hysteresis loop is rectangular, and the coercive field equals the critical field and the anisotropy field. The area of the hysteresis loop represents the upper limit of the SAR for a given material. In real cases of randomly oriented MNPs, the shape of the hysteresis loop is far from a square. For the sake of simplicity, it was proposed [10] to simply express the SAR as the maximum theoretical value provided by the above equation multiplied by a dimensionless coefficient α with values between 0 and 1, with a value of 1 corresponding to an ideal square case where all MNPs have magnetization along the easy axis of magnetization parallel to the magnetic field. Values as high as 0.46 for α were obtained for aligned magnetosomes [49]. In the case of an intermediate regime, none of the above models can be applied, and numerical methods are usually employed [50,51].

For the MNPs synthesized in the present study, due to their large sizes, the heat dissipation mechanism that might have eventually been involved was either Brown relaxation or magnetization reversal. For practical applications of MNPs in clinical hyperthermia, Brownian losses are less relevant for several reasons: Brownian motion is strongly dependent on MNP hydrodynamic volume, which in turn is strongly dependent on the degree of aggregation of MNPs. Moreover, the bonding of MNPs to different biological structures and their agglomeration in endosomes will strongly affect heat dissipation through the Brownian relaxation mechanism. Therefore, we believe that what is more relevant for hyperthermia application is to study the heating mechanism while the Brownian relaxation mechanism is blocked, which means blocking the physical rotation of the MNPs. This also means that the results obtained for MNPs with blocked rotation ensure that the heat released is the same whether the MNPs are individually free or part of an aggregate. In this sense, our experimental setup included, apart from the determination of SAR values in water (the heating curves are presented in Figure S3), the same measurements in a solid matrix. For this purpose, the MNPs were dispersed at 80 °C in liquid PEG 8K, and the samples were cooled, frozen at 60 °C, and cooled down to room temperature. In addition, the heating curves (Figures S4 and S5) were followed only up to 50 °C to ensure that the samples maintained their solid state.

**Figure 3:**
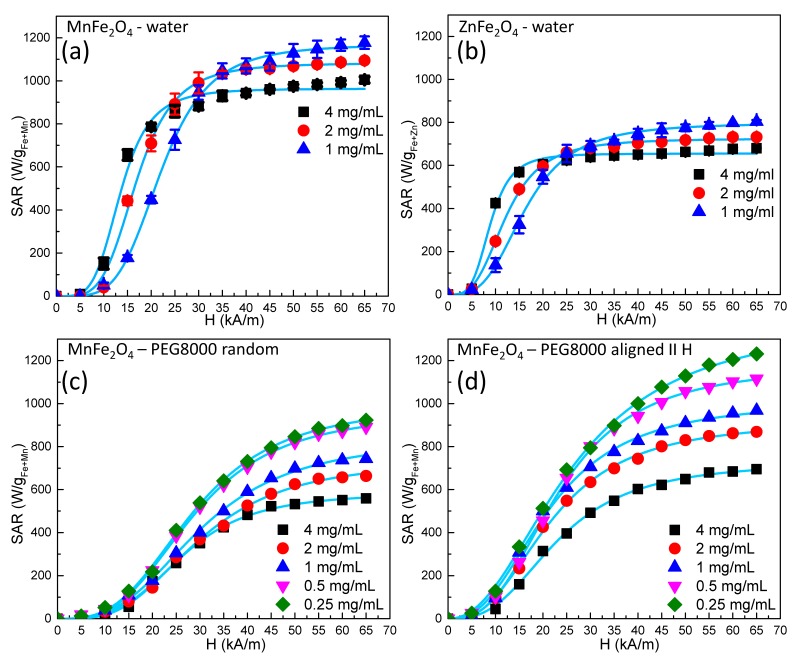
Specific absorption rate (SAR) dependence on the alternating magnetic field amplitude at different MNP concentrations for (**a**) manganese ferrite in water, (**b**) zinc ferrite in water, (**c**) manganese ferrite in solid polyethylene glycol 8000 (PEG 8K) with a random orientation, and (**d**) manganese ferrite in a solid PEG 8K prealigned parallel with alternating magnetic field directions.

Curves representing SAR dependence on the external alternating magnetic field amplitude for a fixed frequency of 355 kHz and for different concentrations (for both Mn and Zn ferrites) are shown in Figure 3.

For all samples, we noticed a saturation of the SAR values as the amplitude of the AC magnetic field increased, with the experimental data being very well fitted by a sigmoidal function. These results are in agreement with the numerical simulation performed by Carrey et al. [10] and Christiansen et al. [51]. The explanation for this behavior is related to the *H_c_*. In other words, for an *H* smaller than the *H_c_* (which is unable to reverse the magnetization of the MNPs), no energy absorption took place when the hysteresis loop was a minor one. At AC magnetic field amplitudes surpassing the *H_c_*, when the hysteresis loop was a major one, the energy lost was maximized. As the amplitude of the AC field increased, saturation was reached, and the SAR values attained their maximum value. Our experimental data were well fitted (*R*^2^ > 0.999) phenomenologically with a simple logistic function:

(1) $$SAR=SAR_{max}\frac{{\left(\frac{H}{H_{cHyp}}\right)}^{n}*\propto }{1+{\left(\frac{H}{H_{cHyp}}\right)}^{n}*\propto }$$

with

(2) $$\propto \;=\frac{n+1}{n-1}$$

where *SARmax* represents the saturation value of the SAR, and *H_cHyp_* is the hyperthermia coercive field, which is the value of the AMF magnetic field amplitude for which the function presents the highest slope [10] or the magnetic field for which the first derivative of SAR against the amplitude of the AMF magnetic field presents a maximum [35]. The exponent *n* indicates how steep the dependence of SAR on the amplitude of the AMF magnetic field is, and as *n* is higher, the model is closer to an ideal Stoner–Wohlfarth one [48].

The values of *H_cHyp_* and the exponent *n* are provided in Table 2 for all samples. From a first inspection of the experimental data, it is very obvious that the hyperthermia performance of Mn ferrites was significantly higher (*SAR_max_* = 1170 W/g_Fe+Mn_) compared to the Zn ferrites (*SAR_max_* = 800 W/g_Fe+Zn_). Another important observation for the samples measured in water was that the saturation SAR values increased as the concentration of the MNPs decreased. On the other hand, for the magnetic field amplitudes below saturation, the behavior was reversed, i.e., the SAR values increased as the concentration increased.

The SAR dependence on the concentration of MNPs is a matter of debate. Most papers have reported a decrease in hyperthermia performance as the concentration increased, but there is also a non-negligible number of reports indicating the opposite behavior [52,53]. In most studies, a decrease in SAR with an increase in the MNP concentration has been explained based on dipole–dipole interactions leading to chain formation and subsequent SAR reduction. This decrease in the heating performance as the concentration of the MNPs increases has been seen as a major drawback in the in vivo application of magnetic hyperthermia because in most cases, MNPs have had much better heating performances in vitro when they are uniformly dispersed. In cell cultures, it has been observed that MNPs accumulate and agglomerate in endosomes, and their heating characteristics drastically decline. Deatsch and Evans [53] interpreted this effect by making a distinction between aggregation and agglomeration and also between the two relaxation mechanisms, Brown and Neel. Bianco-Andujar et al. [40] reported that the decrease in the heating performance of MNPs is due to the demagnetizing effect of interparticle interactions and that multicore MNP aggregates exhibit higher SAR values when larger-core MNPs aggregate in smaller complexes than in the opposite situation. In our particular case, we believe that for high-amplitude magnetic fields, the decrease in the *SAR_max_* with increasing concentrations was mainly due to the increase in the dipolar interactions between neighboring particles as the concentration increased. In the low magnetic field regime, the increase in the SAR with increasing concentration might be explained mathematically in terms of the coercive field. Indeed, in the data in Table 2, one can see that, e.g., for the Mn ferrite, the *H_cHyp_* decreased from 20 kA/m at a concentration of 1 mg/mL to 12.2 kA/m at a concentration of 4 mg/mL. As the *H_chyp_* was smaller, the MNPs reached SAR saturation at smaller values of the alternating magnetic field amplitude. This led to a higher SAR at higher concentrations in this field range. The situation was similar for the Zn ferrite. However, this was valid only when the MNPs were dispersed in water. In solid PEG 8K, the coercive fields did not differ significantly at different concentrations, and in all the field ranges, the SAR decreased with increasing concentration. If we corroborate these two observations, we get the following: i) A reduction in the coercive field due to increasing the concentration occurred only in water, where the particles were mobile; and ii) a similar reduction in the coercive field could be observed in particles prealigned in DC magnetic fields before being frozen in solid PEG 8K (see below). We can give a possible phenomenological explanation for this behavior: As the concentration increased, the interaction between the ferromagnetic MNPs led to the formation of small chains (local structuration), leading to an increase in the magnetic anisotropy, with the consequence of an increase in the SAR values. At higher alternating magnetic field amplitudes, these structures were destroyed, and the SAR decreased with increasing concentrations due to the increase in dipolar interactions.

A 20% decrease in the maximum SAR values was obtained when the hyperthermia experiments were performed in cell culture media (Figure S6c,d), as reported earlier for Fe ferrite [35]. This decrease can be explained by the aggregation of the MNPs in the cell culture media. We tested the stability of our MNPs in cell culture medium, and according to the TEM images (Figure S6a,b), the integrity of the MNPs was not affected by the culture medium. However, it can be observed that the MNPs were grouped into clusters, and it seems that they were embedded in a sort of gel. This aggregation led to a decrease in the mean distance between the MNPs and a subsequent increase in their dipolar interactions, with the consequence of a decrease in their heating performances.

As we presented above, it is quite obvious that for biological applications, one must consider the heating capacity of the MNPs while they are completely immobilized, and we measured the SAR for the Zn and Mn ferrite MNPs dispersed in solid PEG 8K. As can be observed from the data presented in Table 2, there was a significant decrease in the SAR values for both ferrites (from 1170 W/g_Fe+Mn_ to 835 W/g_Fe+Mn_ (at a concentration of 1 mg/mL) for the Mn ferrites) and a dramatic decrease of approximately 50% in the case of the Zn ferrites, where the *SAR_max_* dropped from 655 W/g_Fe+Zn_ to 325 W/g_Fe+Zn_ (at 4 mg/mL). For our large MNPs with a crystallite size of around 30 nm and a multicore diameter around 80 nm, Neel relaxation made a negligible contribution to the SAR, and the main mechanisms for heat dissipation were Brown relaxation and a magnetization reversal due to hysteresis. For the immobilized samples, it was obvious that the Brownian mechanism was hampered for both types of MNPs [54,55]. If we calculated the absolute decrease in SAR values for the two ferrites, we could observe that this decrease was almost the same (335 W/g_Fe+Mn_ in the case of Mn ferrites and 330 W/g_Fe+Zn_ in the case of Zn ferrites). Because both MNPs had similar sizes and similar saturation magnetizations, one can presume that they had the same Brown contribution to the SAR of around 330 W/g_Fe+Mn,Zn_. Because in the case of the Zn ferrite MNPs the Brown mechanism contribution to the SAR was almost 50%, blocking Brown relaxation led to a more pronounced relative decrease in the SAR values.

On the basis of the fact that bacteria-secreted magnetic nanoparticles, so-called magnetosomes, have an increased heating performance mainly attributable to their chain organization [49], several groups have studied the effects of MNP-controlled assembly at a nanoscale on hyperthermia properties [56,57,58]. In a similar attempt, we measured the SAR values for the two ferrites with the MNPs aligned in an external DC magnetic field at 80 °C while the samples were in a liquid state, allowing the sample to freeze with the DC magnetic field on, as described in the Supplementary Materials (Figure S7). For the Mn ferrite samples, which were aligned in a DC magnetic field, we noticed a significant increase in the *SAR_max_* values, with a simultaneous reduction in the hyperthermia coercive fields. For example, for a concentration of 0.25 mg/mL for the aligned samples, the *SAR_max_* was 1395 W/g_Fe+Mn_, while for the randomly oriented MNPs, the *SAR_max_* was only 995 W/g_Fe+Mn_. For the same sample, the coercive field dropped from 23 kA/m to 16.6 kA/m. This decrease in the coercive field is very significant and is important in hyperthermia experiments, especially when large MNPs present this SAR saturation effect. For example, in the case of Mn ferrites in the random sample (Figure 3c), to reach a value of 800 W/g_Fe+Mn_ for the SAR, an AC magnetic field with an amplitude of 45 kA/m was needed, while in the case of the aligned sample, the same value of the SAR could be obtained at an AC magnetic field amplitude of only 30 kA/m. This result was more obvious in the case of the Zn ferrites, where the *SAR_max_* values at 4 mg/mL in PEG 8K were 325 W/g_Fe+Zn_ and 330 W/g_Fe+Zn_ (see Figure S5, right panels), which was very close (within the experimental errors). However, while for the random sample the coercive field was 18 kA/m for the aligned one, the coercive field dropped to only 10.3 kA/m. In other words, an SAR value of 250 W/g_Fe+Zn_ could be reached at an AC magnetic amplitude of 45 kA/m for the random sample and only at 25 kA/m for the aligned one. Our data demonstrate clearly that even in the case of immobilized MNPs, their heating performance could be significantly improved and were comparable or even larger than their mobile counterparts if they were aligned in an external DC magnetic field.

In the case of PEG 8K, we were able to measure the SAR values down to a concentration of 0.25 mg/mL, which is close to the concentration limit for intrinsic cytotoxic effects of MNPs (see below). For the case of randomly distributed MNPs (which was the most relevant for the in vitro and in vivo experiments), there was only a very slight change in the *SAR_max_* values, as the concentration decreased from 0.5 mg/mL to 0.25 mg/mL (*SAR_max_* decreased from 995 W/g to 965 W/g), and we considered this to be the limit of *SAR_max_* for low concentrations. In the case of the measurements performed in water, we noticed that at concentrations below 1 mg/mL, the MNPs tended to align along the walls of the measuring vial when exposed to high AC magnetic fields, as has been reported by other groups [31], making it a difficult task to accurately estimate the SAR.

### 3.4. Cytotoxicity Assessment

In vivo applications of MNPs require an evaluation of their in vitro toxicity. Thus, relevant information about the cytotoxicity of both types of MNPs and their cell internalization pathways can be obtained from these types of studies.

**Figure 4:**
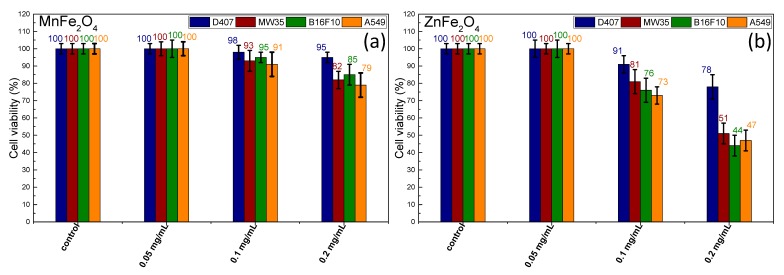
Cell viabilities exhibited by the four cell lines exposed to (**a**) manganese ferrite and (**b**) zinc ferrite MNPs (mean ± (SEM), *n* = 3) for three different concentrations.

In this regard, a standard MTT assay was performed on four different cell lines at three concentrations of MNPs: 0.05, 0.1, and 0.2 mg/mL. The cultured cells were incubated for 24 h at 37 °C in the same conditions. As observed under an inverted optical microscope, upon incubation the cells did not show any sign of cell suffering: they were still confluent in the culture flask, and they were not detached. Neither type of MNP exhibited cytotoxicity at the lowest MNP concentration of 0.05 mg/mL, as indicated in Figure 4.

Upon an increase of the MNP concentration to 0.1 mg/mL, the cellular viability of all types of cells exhibited a negligible decrease in the case of MnFe_2_O_4_ MNPs (98–91%). On the contrary, the cellular viability was sufficiently affected by the presence of ZnFe_2_O_4_ MNPs (91–73%) once their concentration was increased to 0.1 mg/mL. A 50% decrease in cellular viability was recorded when the concentration of ZnFe_2_O_4_ MNPs was further increased to 0.2 mg/mL. At this level of concentration, the cytotoxic effects of MnFe_2_O_4_ MNPs started to grow, producing a decrease in cell viability (5–20%).

Consequently, these results show a higher toxicity of the ZnFe_2_O_4_ MNPs compared to the MnFe_2_O_4_ MNPs or the large Fe_3_O_4_ MNPs [35]. The previous results obtained by our group for large Fe_3_O_4_ MNPs synthesized in EG revealed a viability of 80% for D407 cells and between 88% and 90% for the other three cell lines [35]. The cytotoxic profile of MnFe_2_O_4_ MNPs was 10% more pronounced than the one recorded for large Fe_3_O_4_ MNPs [35]. It should be noted that the cancer cells were more affected by the cytotoxic profile of both MNPs in comparison to the normal cells. Among the cancer cells, the A549 cell type exhibited the highest sensitivity to both MNPs at all three concentrations, followed by the B16F10 line and the MW35 line.

Thus far, it has been proven that Mn ferrite MNPs with sizes below 30 nm do not induce any cytotoxicity effects on different cancer cell lines at concentrations exceeding 0.2 mg/mL [59,60], yet they are capable of inducing cellular apoptosis in MH experiments. In our case, the cytotoxicity profile at 0.2 mg/mL could be attributed to the larger size of the MNPs and the lack of a protecting biocompatible coating. On the contrary, it has been found that spherical Zn ferrite MNPs with an average size of 44 nm induce dose-dependent cytotoxicity and oxidative stress in different types of cells in the dosage range of 0.01–0.04 mg/mL [61]. A mild in vitro toxicity has been reported for Zn ferrite MNPs 11 nm in size at doses of less than 100 mg/mL [62]. Moreover, it has also been found that Mn ferrite MNPs doped with Zn^2+^ ions produce a considerable cytotoxicity profile in human endothelial cells at doses of up to 0.1 mg/mL [63] unless these types of MNPs are protected by a polyethylene glycol layer [64]. Given these results, it is quite clear that the high cytotoxic profile of our Zn ferrite MNPs might be due not to the intrinsic toxic nature of Zn but rather to the presence of the ZnO phase, which has been proven to display a strong cytotoxic effect on cancer cells [65].

### 3.5. Cell Uptake Properties

The TEM examination of the cultured cells after 4 h of incubation revealed the ability of both normal and cancer cell lines to internalize the two types of MNPs (MnFe_2_O_4_ and ZnFe_2_O_4_).

**Figure 5:**
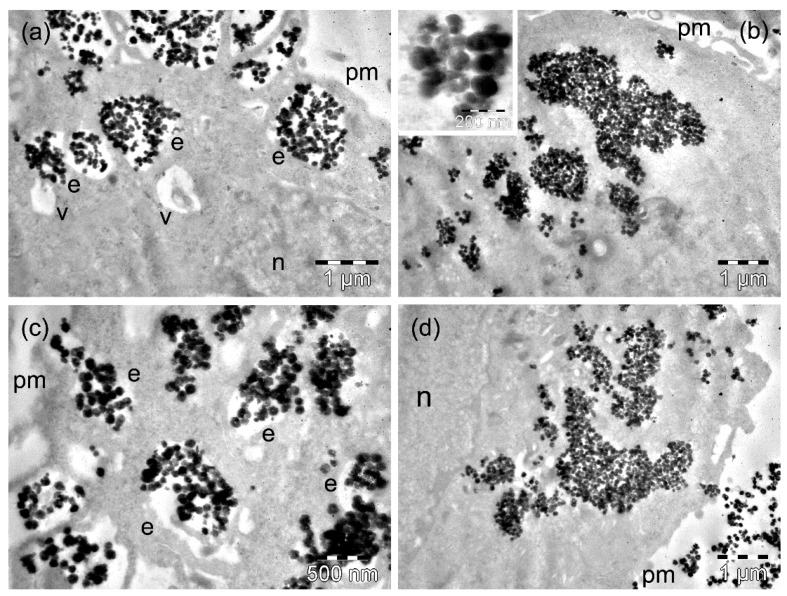
TEM images of D407 cells containing MnFe_2_O_4_ MNPs after 4 h (**a**,**b**) and 24 h (**c**,**d**) of incubation time. The letters n, e, v, and pm denote the nucleus, the endosomes, the vacuoles, and the plasma membranes, respectively. The inset in (b) on the left shows the formation of an endosome with MnFe_2_O_4_ MNPs.

After 4 h of incubation of D407 cells with MnFe_2_O_4_ MNPs, the MNPs were present in high amounts within the cytoplasm. On the one hand, many MnFe_2_O_4_ MNPs were still included in large or very large endosomes located generally in the proximity of the plasma membrane (Figure 5a). At the cell periphery, more MNPs appeared, surrounded by thin cellular extensions that were captured (Figure 5a). On the other hand, important amounts of MnFe_2_O_4_ MNPs were observed as large clusters or were already dispersed, as they were in contact with the cytosol (Figure 5b). Once they arrived at the cytosol, some of these MNPs showed a tendency to disintegrate into smaller fragments (inset of Figure 5b). This aspect was observed inside of a few endosomes as well (not shown). Rare vacuoles were also identified in the cells containing important amounts of MNPs (Figure 5a), but some of these vacuoles could have been endosomes with a reduced number of MNPs. At 24 h, a low number of D407 cells continued the process of endocytosis of MnFe_2_O_4_ MNPs, which was suggested by the presence of numerous large endosomes filled with MNPs (Figure 5c) and was confirmed by the measurements performed (Table S1). Most of the cells showed individual MNPs dispersed within the whole cytosol (including in the immediate proximity of the nucleus), but they mainly contained smaller or bigger groups of MNPs with cytosolic locations (Figure 5d). Among the studied cells, only a few displayed a tendency for vacuolation (Figure 5c, Table S2).

**Figure 6:**
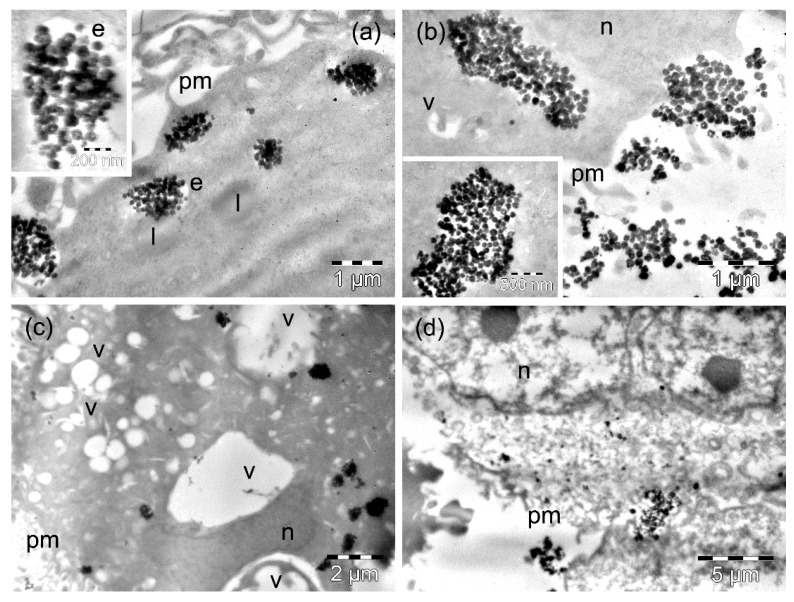
TEM images of MW35 cells containing MnFe_2_O_4_ MNPs after 4 h (**a**,**b**) and 24 h (**c**,**d**) of incubation time. The letters n, e, v, l, and pm denote the nucleus, the endosomes, the vacuoles, the lipid droplets, and the plasma membranes, respectively. The inset in (**a**) on the left shows the formation of an endosome with Mn_2_Fe_3_O_4_ MNPs.

After 4 h of incubation of MW35 cells with MnFe_2_O_4_ MNPs, we found only rare endosomes loaded with MNPs, especially at the periphery of the cells, and they contained relatively low amounts of MNPs (Figure 6a). The majority of the MnFe_2_O_4_ MNPs internalized by the cells in this line were visible as smaller (Figure 6a) or larger clusters (Figure 6b). Rare vacuoles were observed in the cells in this experimental condition (Figure 6b), and also a few dead cells (not shown). The amount of internalized MnFe_2_O_4_ MNPs remained low at 24 h (Table S1): they were mainly present as small clusters in the cytosol (Figure 6c). The cytoplasmic vacuolation recorded for the cells (preserving their integrity) was extensive (Figure 6c, Table S2). Many other cells died: they displayed polymorphous nuclei with a reduced amount of compacted chromatin, a rarefied and electron-lucent cytoplasm, without visible organelles, and a disrupted plasma membrane (Figure 6d). In some of the dead cells, dispersed MNPs were visible among the debris (Figure 6d).

Malignant cells from the B16F10 line reacted to incubation with MnFe_2_O_4_ MNPs similarly (in general) to the cells from the MW35 line both at 4 h (Figure S8a,b) and at 24 h (Figure S8c,d), but a higher amount of internalized MNPs was measured after the short incubation time (Table S1). However, they seemed to be slightly less sensitive to the toxic effect of the MNPs: the cells had a reduced number of vacuoles, especially after 24 h of incubation (Table S2), and a lower number of dead cells was recorded at 24 h as well.

The cells from the line A549 showed the highest ability to internalize the MnFe_2_O_4_ MNPs that were found in very large amounts in the endosomes and that were free at the level of cytosol, both after 4 h (Figure S9a,b, Table S1) and after 24 h (Figure S9c,d) of incubation. An ANOVA revealed significant differences compared to the surface areas of the cells occupied by these MNPs and by the others (*p* < 0.05 in each case) after 4 h of incubation. At 24 h, we also obtained statistically significant differences (*p* < 0.05) for the ability of this cell type to internalize MnFe_2_O_4_ MNPs as compared to MW35 and B16F10 cells. In many cells, large MNPs containing endosomes seemed to fuse, generating very large vesicles (Figure S9a). At 4 h, many cytoplasmic vacuoles were also present in the cells (Figure S9a, Table S2), while at 24 h the majority of cells suffered profound alterations (Figure S9d), with many being destroyed by the toxic effect of the MNPs (inset of Figure S9d).

MW35 cells manifested a much-reduced capacity to internalize the MnFe_2_O_4_ MNPs. However, the observation of several dead cells consecutive to the 4 h of incubation suggested that these cells possessed a reduced resistance to the toxic effects of the MNPs compared to the normal epithelial cells.

**Figure 7:**
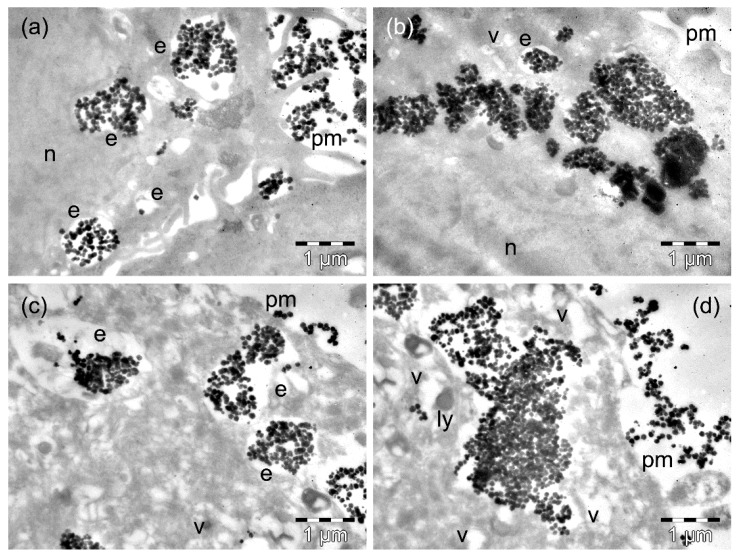
TEM images of D407 cells containing ZnFe_3_O_4_ MNPs after 4 h (**a**,**b**) and 24 h (**c,d**) of incubation time. The letters n, e, v, ly, and pm denote the nucleus, the endosomes, the vacuoles, the lysosomes, and the plasma membranes, respectively.

The D407 cells that were incubated for 4 h with the ZnFe_2_O_4_ MNPs contained a comparable amount of MNPs (as in the case of incubation with MnFe_2_O_4_ MNPs: Table S1), both when still packed into endosomes next to the plasma membrane (Figure 7a) and as large clusters in the cytosol (Figure 7b). Many cells were deeply affected by the presence of the MNPs, and others were destroyed (not shown). After 24 h of incubation, the D407 cells showed MNPs with endosomal (Figure 7c, Table S2) and cytosolic (Figure 7d) locations. Most of these cells presented with extensive vacuolation in their cytoplasm (Figure 7c), while others (in high numbers) had a rarefied cytoplasm and disrupted the plasma membrane (Figure 7d).

**Figure 8:**
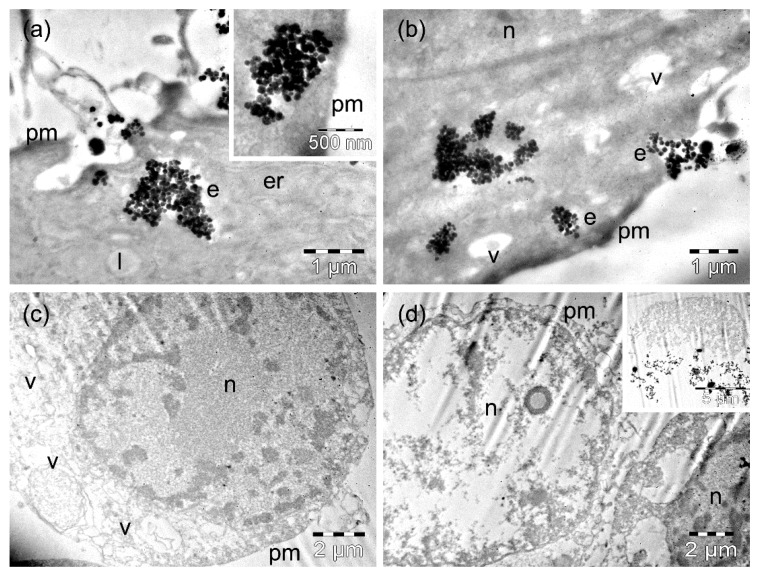
TEM images of MW35 cells containing ZnFe_3_O_4_ MNPs after 4 h (**a**,**b**) and 24 h (**c,d**) of incubation time. The letters n, e, v, l, and pm denote the nucleus, the endosomes, the vacuoles, the lipid droplets, and the plasma membranes, respectively.

The MW35 cells incubated for 4 h with ZnFe_2_O_4_ MNPs contained a relatively low amount of MNPs compared to incubation with MnFe_2_O_4_ MNPs (Table S1). ZnFe_2_O_4_ MNPs were rarely found in endosomes (in general) of reduced size that were located next to the plasma membrane (Figure 8a,b). Endosomes with a disrupted membrane (Figure 8a) released their MNP cargo very quickly, with groups of internalized MNPs (no longer surrounded by membranes) being observed inside the cells in the vicinity of the plasma membrane (inset of Figure 8a). More numerous MNPs were observed grouped in various regions of the cytosol (Figure 8b). Many cells contained a few (Figure 8b) or numerous (not shown) vacuoles (Table S2). This finding did not exclude the presence of viable cells in the culture after a longer incubation time, but it is very likely that a thin layer of such intact cells (and the densest ones as well) was removed from the blocks containing the cell pellets during the trimming procedure. It also remains possible that many fragilized cells (more sensitive after the incubation with MNPs) were damaged during centrifugation. The cells still visible showed a nucleus containing clumped chromatin, a vacuolated cytoplasm, and disrupted plasma membranes (Figure 8c), or they were fragmented (Figure 8d). The lowest amount of MNPs was identified within the cellular debris (Table S1), while vacuoles occupied the largest area of the damaged cell cytoplasm (statistically significant at *p* < 0.05) compared to 4 h of incubation, but in the other types of cells incubated with ZnFe_2_O_4_ MNPs (Table S2).

The malignant cells from the B16F10 line proved to be more sensitive to the toxicity of the ZnFe_2_O_4_ MNPs than to that of the MnFe_2_O_4_ MNPs. These cells responded strongly to the ZnFe_2_O_4_ MNPs accumulated in endosomes and the cytosol (Figure S10a), and after a short incubation time, the cytoplasm of many cells showed extensive vacuolation and cytoplasmic rarefaction (Figure S10b, Table S2). In the samples photographed after 24 h of incubation, almost all the cells were dead, and the debris of the cells was visible, within which were important amounts of MNPs that were still present (Figure S10c,d). The B16F10 cells internalized the highest amounts of MNPs at 24 h (Table S1), but this was statistically significant only when compared to the MNPs internalized by the MW35 cells (*p* < 0.05).

The cells from line A549 reacted almost identically to the malignant cells from the previous line, both at 4 h (Figure S11a,b), and at 24 h (Figure S11c,d) of incubation with the ZnFe_2_O_4_ MNPs. However, a particularity was noted: the MNP-loaded endosomes were extremely rare in these cells, with the majority of MNPs being grouped or dispersed within the cytosol. An ANOVA showed significantly higher values when compared to the level of vacuolization in this cell type in the results recorded for the same cells after 24 h of incubation with MnFe_2_O_4_ MNPs (*p* < 0.05).

No MNPs were found in the lysosomes: neither MnFe_2_O_4_ MNPs nor ZnFe_2_O_4_ MNPs in the cells of the four studied lines. This aspect is important to mention since other metallic NPs—Ag [66] and Au [67,68]—have been reported as accumulating inside lysosomes, and this way the cells could limit and/or prevent their toxic effects. In the tested cancer cells, many endosomes were surrounded by a discontinued membrane. This ultrastructural aspect indicated an accentuated fragility of membranes in the three types of malignant cells that in turn facilitated the release of the MNPs into the cytosol. The early presence of vacuoles after 4 h of incubation was indicative of the cellular sufferance induced by the MNPs, especially by the ZnFe_2_O_4_ MNPs.

Compared to incubation with MnFe_2_O_4_ MNPs, all of the studied cell lines internalized the ZnFe_2_O_4_ MNPs in lower amounts, and they also were more sensitive to these ZnFe_2_O_4_ MNPs. The human melanoma cells presented a lower capacity to internalize both types of MNPs compared to the normal epithelial cells but also compared to the malignant cells in the other lines. The behavior of the cells also had some particularities determined by the cell line, but especially by the type of MNP.

## 4. Conclusions

Manganese and zinc ferrite MNPs were synthesized using a polyol-based method and were systematically investigated and compared for their structural, magnetic, hyperthermia, cytotoxic, and cell uptake properties. For both ferrite classes, the TEM and XRD data suggested a multicore polycrystalline structure approximately 80 nm in diameter composed of several individual crystallites of ~30 nm. Both the Mn and Zn ferrite MNPs synthesized in EG displayed a high *M_s_* of 90 emu/g and 95 emu/g for Mn and Zn, respectively, values which were significantly higher than the M_s_ of Fe_3_O_4_ MNPs synthesized in EG (75 emu/g) in the same conditions.

For both classes of MNPs, a sigmoidal dependence of the SAR = *f*(*H*) was detected, which was very well fitted with a simple logistic function, with *SAR_max_* values 50% higher for Mn ferrite compared to Zn ferrite. At high external alternating magnetic field amplitudes, near saturation, the SAR values in water decreased when the concentration of the MNPs increased, most probably due to an increase in the dipolar interactions between the MNPs as the concentration increased. At lower external alternating magnetic field amplitudes, an opposite phenomenon was observed, where increasing the concentration led to an increase in SAR. When the MNPs were immobilized in a solid matrix, a significant decrease in the *SAR_max_* was measured, and this behavior was attributed to the blocking of the Brownian viscous relaxation mechanism. Interestingly, the drop in the SAR values was almost the same (~330 W/g_Fe+Mn,Zn_) for the two classes of MNPs, although this decrease was ~50% in the case of Zn ferrites and ~25% in the case of Mn ferrites. Because both types of MNPs had similar sizes, structures, and saturation magnetizations, this result suggests that the Brownian contribution to the SAR depended mainly on these parameters.

When the MNPs were aligned in a uniform DC magnetic field prior to their immobilization in PEG 8K, we noticed a significant increase in *SAR_max_*, with values of 1395 W/g_Fe+Mn_ for Mn ferrites (for a concentration of 0.25 mg/mL), which were even higher than the *SAR_max_* measured in water for the same type of MNPs. Simultaneously, a significant decrease in the hyperthermia coercive field was observed, meaning that the same SAR could be obtained at a significantly lower magnetic field amplitude in the aligned sample. In the case of the Zn ferrites, a very slight increase in the SAR was obtained for the aligned sample, and a significant decrease in the hyperthermia coercive field was detected.

Our results clearly demonstrate that the polyol-based method can be successfully used for the synthesis of high-crystallinity ferrites with remarkable high-saturation magnetizations and enhanced SAR values. Moreover, our results, which were obtained with immobilized and aligned samples, open new ways for finding methods to improve the heating performances of MNPs in real applications.

The MTT assays performed on the four cell lines revealed a very small level of toxicity for Mn ferrite MNPs, in accordance with other published data for the range of concentrations used (up to 0.2 mg/mL). However, in the case of the Zn ferrites for the same range of concentrations used, ~50% were found dead after 24 h of incubation. This higher toxicity for the Zn ferrite might be because of the formation of a ZnO phase, which is known to be toxic for most cell lines. The cellular uptake experiments showed that both MNPs penetrated the cells through endocytosis in a time-dependent manner. In the tested cancer cells, many endosomes were surrounded by a discontinued membrane, indicating an accentuated fragility of membranes in the three types of malignant cells that in turn facilitated the release of the MNPs into the cytosol. All of the studied line cells were more sensitive to the ZnFe_2_O_4_ MNPs compared to incubation with the MnFe_2_O_4_ MNPs.

## Figures and Tables

**Table 1:** Magnetic properties of MnFe_2_O_4_ and ZnFe_2_O_4_ compared to Fe_3_O_4_ (synthesized in EG).

Sample	4 K				300 K				*K_eff_* (·10^4^ J/m^3^)
	*M_s_* (emu/g)	*H_c_* (kA/m)	*M_r_* (emu/g)	*M_r_*/*M_s_*	*M_s_* (emu/g)	*H_c_* (kA/m)	*M_r_* (emu/g)	*M_r_*/*M_s_*	
MnFe_2_O_4_	90	18	20	0.22	76	5	6	0.08	1.1
ZnFe_2_O_4_	95	26	25	0.26	58	12	16	0.28	1.7
Fe_3_O_4_^1^	75	27	21	0.28	68	6	7	0.10	1.4

^1^ The data are reproduced with permission from Reference [35].

**Table 2:** Fitting results of SAR evolution with AMF magnetic field amplitude.

Sample	Conditions	*C* (mg/mL)	*SAR_MAX_* (W/g_Fe+Mn, Zn_)	*H_cHyp_* (kA/m)	Power Coefficient *n*
MnFe_2_O_4_	Water	4	965 ± 15	12.2 ± 0.4	4.4 ± 0.5
		2	1080 ± 11	15.2 ± 0.3	4.3 ± 0.3
		1	1170 ± 9	20.0 ± 0.2	4.4 ± 0.2
	PEG 8K, randomly distributed	4	585 ± 8	22.5 ± 0.4	3.6 ± 0.2
		2	735 ± 18	24.3 ± 0.7	3.1 ± 0.2
		1	835 ± 18	24.0 ± 0.6	3.00 ± 0.1
		0.5	965 ± 10	23.3 ± 0.3	3.1 ± 0.1
		0.25	995 ± 16	23.0 ± 0.5	3.0 ± 0.1
	PEG 8K-aligned parallel with H	4	730 ± 11	17.8 ± 0.4	2.8 ± 0.1
		2	920 ± 14	16.0 ± 0.4	2.6 ± 0.1
		1	1015 ± 20	15.2 ± 0.5	2.5 ± 0.1
		0.5	1180 ± 11	17.8 ± 0.2	2.8 ± 0.1
		0.25	1395 ± 17	16.6 ± 0.4	2.2 ± 0.1
ZnFe_2_O_4_	Water	4	655 ± 7	8.00 ± 0.3	4.1 ± 0.5
		2	725 ± 4	10.0 ± 0.2	3.2 ± 0.1
		1	800 ± 8	13.5 ± 0.3	3.3 ± 0.2
	PEG 8K, randomly distributed	4	325 ± 8	18.0 ± 0.7	2.4 ± 0.1
	PEG 8K-aligned parallel with H	4	330 ± 3	10.3 ± 0.2	2.4 ± 0.1

## Supplementary Materials

The following are available online at https://www.mdpi.com/2079-4991/9/10/1489/s1, Figure S1: ZFC and FC magnetization curves of MnFe_2_O_4_ and ZnFe_2_O_4_ MNPs acquired in an external magnetic field of 50 mT; Figure S2: ATR-FTIR spectra for TMAOH and TMAOH coated manganese ferrite MNPs; Figure S3: Heating curves of MnFe_2_O_4_ and ZnFe_2_O_4_ MNPs dispersed in water at different concentrations, recorded as a function of AMF amplitudes at 355 kHz; Figure S4: Heating curves of MnFe_2_O_4_ dispersed in PEG 8K at different concentrations either randomly or prealigned in a static magnetic field, recorded as a function of AC magnetic field amplitudes at 355 kHz; Figure S5: Heating curves of ZnFe_2_O_4_ dispersed in PEG 8K either randomly or prealigned in a static magnetic field and SAR values of ZnFe_2_O_4_ MNPs dispersed in PEG 8K either randomly or prealigned in a static magnetic field as a function of AC field amplitudes and at a frequency of 355 kHz; Figure S6: Large-scale (a) and zoomed-in (b) TEM images of MnFe2O4 MNPs dispersed in cell culture medium and SAR dependence on the alternating magnetic field amplitude for manganese ferrite (c) and zinc ferrite (d) in water and cell culture medium at a concentration of 4 mg/ML; Figure S7: Magnetic induction calibration curve between two neodymium (Ne–Fe–B) magnets situated 7 cm from each other, which were used to align the MNPs in a solid matrix before the hyperthermia experiments; Figure S8: TEM images of B16F10 cells containing MnFe_2_O_4_ MNPs after incubation for 4 h and 24 h; Figure S9: TEM images of A549 cells containing MnFe_2_O_4_ MNPs after incubation for 4 h and 24 h; Figure S10: TEM images of B16F10 cells containing ZnFe_2_O_4_ MNPs after incubation for 4 h and 24 h; Figure S11: TEM images of A549 cells containing ZnFe_2_O_4_ MNPs after incubation for 4 h and 24 h; Table S1: Surface area (in µm^2^/cell) occupied by MNPs in the four types of cells; Table S2: Surface area (in µm^2^/cell) occupied by cytoplasmic vacuoles in the four types of cells.
