# Supplementary material for: Hyperthermia, Cytotoxicity, and Cellular Uptake Properties of Manganese and Zinc Ferrite Magnetic Nanoparticles Synthesized by a Polyol-Mediated Process
Source: Nanomaterials (Basel). 2019 Oct 18;9(10):1489. doi: 10.3390/nano9101489 (PMC6835619; doi:10.3390/nano9101489)
Supplement: Supplementary file 1 [file nanomaterials-09-01489-s001.pdf]

Supplementary Materials

Hyperthermia, Cytotoxicity, and Cellular Uptake Properties of Manganese and Zinc Ferrite Magnetic Nanoparticles Synthesized by a Polyol-Mediated Process

Cristian Iacovita [1], Adrian Florea [2], Lavinia Scorus [1], Emoke Pall [3], Roxana Dudric [4], Alin Iulian Moldovan [5], Rares Stiufiuc [1,5], Romulus Tetean [4] and Constantin Mihai Lucaciu [1,*]

[1] Department of Pharmaceutical Physics-Biophysics, Faculty of Pharmacy, "Iuliu Hatieganu" University of Medicine and Pharmacy, Pasteur 6, 400349 Cluj-Napoca, Romania; cristian.iacovita@umfcluj.ro (C.I.); scorus.lavinia@gmail.com (L.S.); rares. stiufiuc@umfcluj.ro (R.S.);

[2] Department of Cell and Molecular Biology, Faculty of Medicine, "Iuliu Hațieganu" University of Medicine and Pharmacy, Pasteur 6, 400349 Cluj-Napoca, Romania; aflorea@umfcluj.ro

[3] Department of Reproduction Obstetrics and Veterinary Gynecology, University of Agricultural Sciences and Veterinary Medicine, Manastur 3-5, 400372 Cluj-Napoca, Romania; pallemoke@gmail.com

[4] Faculty of Physics, "Babes Bolyai" University, Kogalniceanu 1, 400084 Cluj-Napoca, Romania; roxana.pacurariu@phys.ubbcluj.ro (R.D.); romulus.tetean@phys.ubbcluj.ro (R.T.)

[5] Department of Bionanoscopy, MedFuture Research Center for Advanced Medicine, "Iuliu Hatieganu" University of Medicine and Pharmacy, Pasteur 4-6, 400337 Cluj-Napoca, Romania; alin.moldovan@umfcluj.ro

* Correspondence: clucaciu@umfcluj.ro; Tel.: 004-074-464-7854

1. Specific Absorption Rate (SAR) determination

Experimentally, the specific absorption rate (SAR), called sometimes specific power loss (SLP), is defined as the heat released from a suspension of magnetic nanoparticles (MNPs) in unit time reported to their mass, expressed usually in *W/g*:

$$SAR = \frac{P}{m_{MNP}} = \frac{Q}{\Delta t \; m_{MNP}}$$

the heat is calculated as the equivalent heat needed to increase the sample temperature with the same number of *K* in a given time interval,

$$Q = mc\Delta T$$

where Q is the heat, m is the mass of the sample subjected to is heating in alternating magnetic field, c specific heat of the sample (with a good approximation one can consider specific heat of water for dilute magnetic nanoparticles suspended in water), ΔT is the temperature increase of the sample, and Δt is the time during which the alternating magnetic field was applied.

The MNPs were weighed and then were suspended in different media to the desired concentrations for measurements of their hyperthermia properties. An amount of 0.5 ml of each sample was placed in round bottom test tubes with a total 2 ml capacity which was then positioned in the center of the coil. In order to preserve a constant temperature environment inside the coil, the test tubes were surrounded by a low diameter tubing through which water has been circulated at the desired temperature. Care was taken for the samples to be placed in the same position each time inside the coil to be subject to the same field strengths. The temperature was measured with an optical fiber thermocouple connected to a computer which allows temperature measurement every second. Note that fiber optics thermocouple provides precise temperature measurement without being influenced by the magnetic field. Every time the optical fiber thermocouple was placed in the middle of the volume occupied by the aqueous solution of magnetic nanoparticles. The samples dispersed in

water were measured at least 3 times and the samples were sonicated for 30 seconds before the measurement in order to ensure uniform dispersion of the particles in the suspension medium. Since the hyperthermia measurements were performed in a non-adiabatic environment, we have taken into account only the linear portion of the time dependence of the temperature (see black rectangles in both figures SI2 and SI3), for the SAR determination. In the SAR calculation we have considered for water: ϱ = 1 g/ml, c = 4186 J/kg K; for PEG8K: ϱ = 1.0852 g/ml, c = 2135.27 J/kg K. Magnetic field calibration was performed as described in ref. [27] from the main paper.

2. Cell lines and cell culture details.

Human melanoma MW35 cells were cultured in RPMI 1640 medium supplemented with 10% fetal bovine serum (Hyclone), 1 mM glutamine (Sigma-Aldrich), 1% antibiotic-antimycotic 100x (Sigma-Aldrich). The mouse melanoma cell line B16F10 was maintained in DMEM (Sigma-Aldrich) medium supplemented with 10% fetal bovine serum (Hyclone), 1 mM glutamine (Sigma-Aldrich), 1% antibiotic antimycotic 100x (Sigma-Aldrich). Human lung adenocarcinoma A549 cells were grown in RPMI 1640 (Sigma-Aldrich) medium with 1% Antibiotic-antimycotic 100x (Sigma-Aldrich) and 10% FBS (Hyclone). Human retinal pigment epithelial D407 cells were cultured in DMEM high glucose (Sigma-Aldrich) medium supplemented with 10% fetal bovine serum (Hyclone), 1 mM glutamine (Sigma-Aldrich), 1% antibiotic-antimycotic 100x (Sigma-Aldrich). Cultures were maintained at 37°C in 5% CO_2 and 95% relative humidity.

3. Cellular uptake assessment

They were prefixed directly in the culture flask with 2.7% glutaraldehyde (Electron Microscopy Sciences, Hatfield, USA) in 0.1M phosphate buffer (pH 7.4) at 4°C for 1.5 h. After centrifugation at 1500 RPM for 10 min the cells were washed four times with 0.1M phosphate buffer (pH 7.4), and then post-fixed for 1.5 h with 1.5% osmium tetroxide (Sigma-Aldrich, St. Louis, USA) at 4°C. They were next dehydrated in acetone series (30% to 100%), infiltrated and embedded in Epon 812 resin (Fluka, Buchs, Switzerland). The blocks polymerized for 72 hours at 60°C were trimmed and cut with glass knives on a Bromma 8800 ULTRATOME III (LKB, Stockholm, Sweden). The ultrathin sections (60-80 nm) were collected on 3 mm copper grids (with formvar film) and contrasted for 7 min with uranyl acetate (Merck, Billerica, USA). The samples were examined on the JEOL-JEM 1010 transmission electron microscope (Jeol Ltd., Tokyo, Japan), equipped with a Mega VIEW III camera (Olympus, Soft Imaging System, Münster, Germany) and operating at 80 kV. In order to quantitatively assess the internalized MNPs and of the level of cytoplasmic vacuolization we used a CellD software (Olympus Soft Imaging Solutions GMBH, Münster, Germany). On the recorded images, manual measurements of the cellular surface occupied by the MNPs (n between 9-220) and by the vacuoles (n between 23-302) respectively were performed in each cell (using the 3-point circle tool). The obtained values, expressed as mean ± standard error, were subjected to analysis of variance (one-way ANOVA, $p<0.05$ considered significant). The results from the 16 groups were compared using a post hoc Tukey test (significant at $p < 0.05$). Origin 7SR1 software (OriginLab Corporation, Northampton, USA) was used for the statistical analysis.

4. Field Cooled and Zero Field Cooled curves for the two types of MNPs

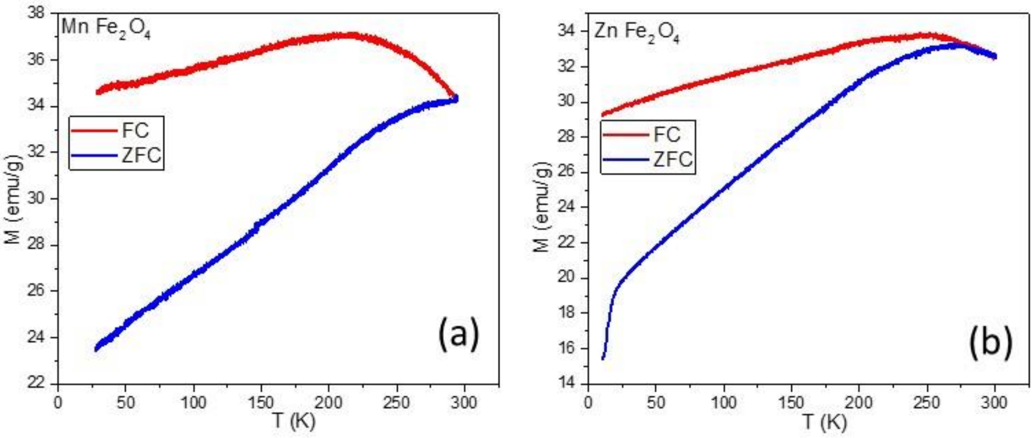

Figure S1. ZFC and FC magnetization curves of **(a)** $MnFe_2O_4$ **(b)** $ZnFe_2O_4$ MNPs, acquired in an external magnetic field of 50 mT.

5. FTIR spectrA, evidencing the TMAOH coating of the MNP

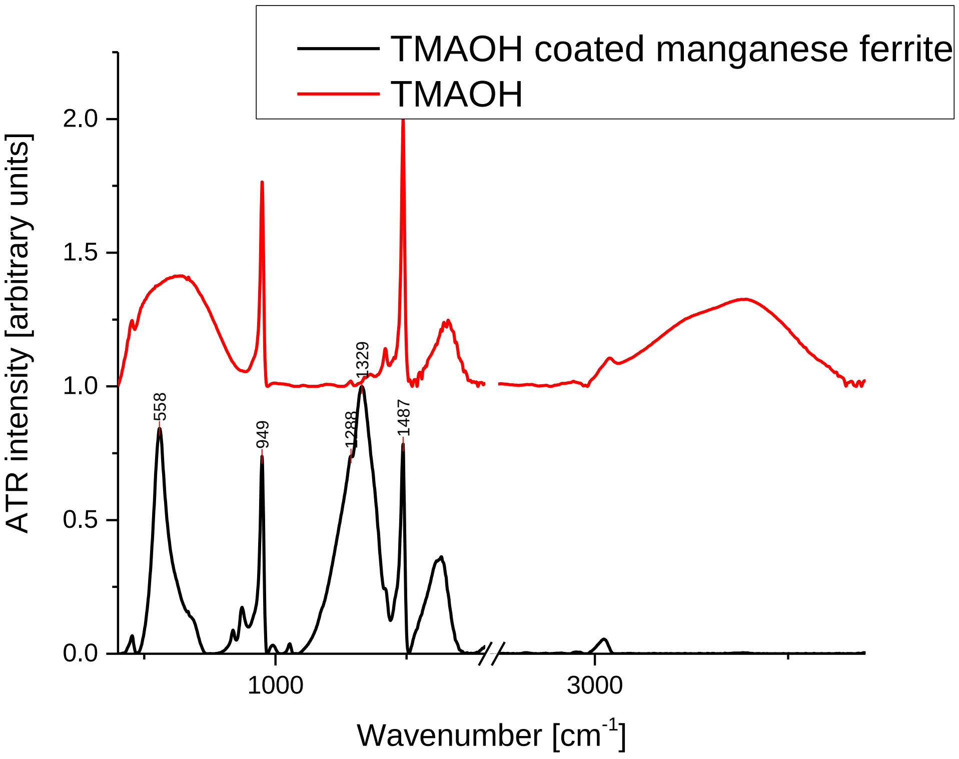

Figure S2. ATR-FTIR spectra for TMAOH and TMAOH coated manganese ferrite MNPs. The typical bands of TMAOH are: the υ_{asym} (C–N at 949 cm^{-1}) and asymmetric methyl deformation mode, $\delta_{asym}(CH_3)$ at 1487 cm^{-1} [1] can be clearly observed on the TMAOH coated MNPs.

6. Heating curves T = f(time) curves for the two types of MNPs dispersed in water

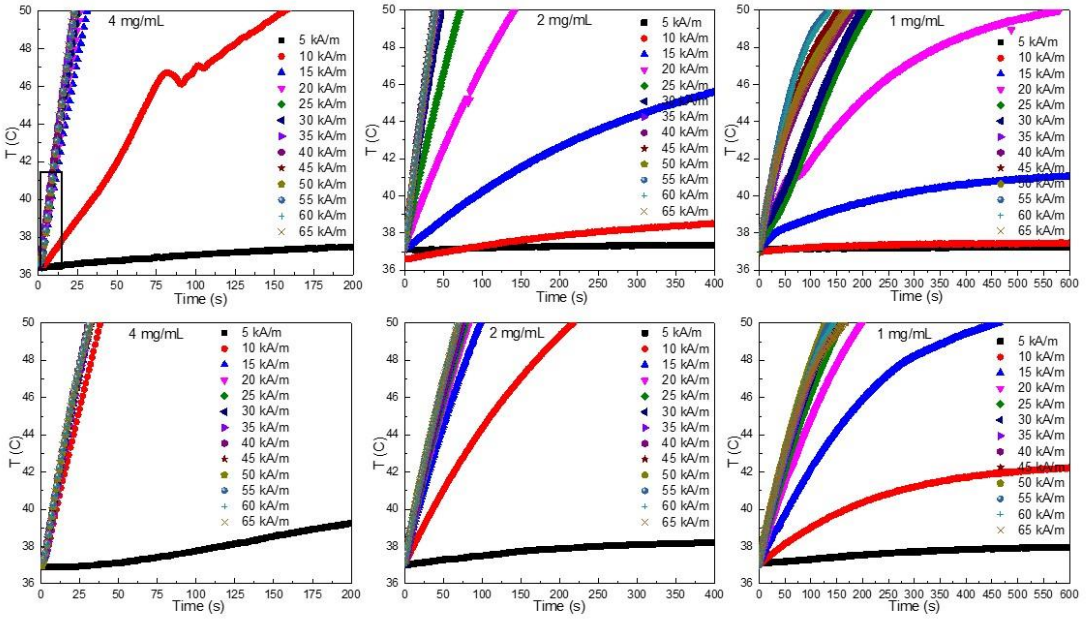

Figure S3. Heating curves of $MnFe_2O_4$ (upper row) and $ZnFe_2O_4$ (lower row) MNPs dispersed in water at different concentrations, recorded as a function of AC magnetic field amplitudes at 355 kHz. The black rectangles frame the slope ($\Delta T/\Delta t$) used in SAR determination.

7. Heating curves T = f(time) curves for the two types of MNPs dispersed in PEG8K

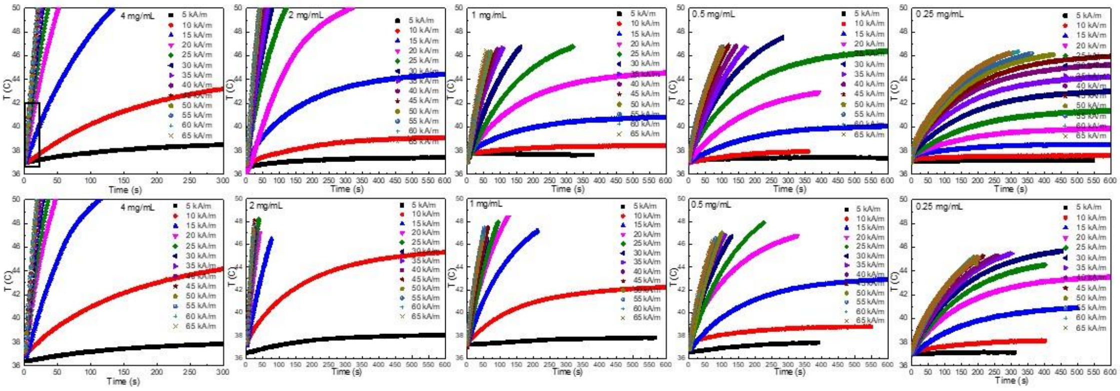

Figure S4. Heating curves of $MnFe_2O_4$, dispersed in PEG 8K at different concentrations either randomly (upper row) or pre-aligned in a static magnetic field (lower row), recorded as a function of AMF amplitudes at 355 kHz. The black rectangles frame the slope ($\Delta T/\Delta t$) used in SAR determination.

8. Heating curves T = f(time) and SAR = f(H) curves for the $ZnFe_2O_4$ MNPs dispersed in PEG8K

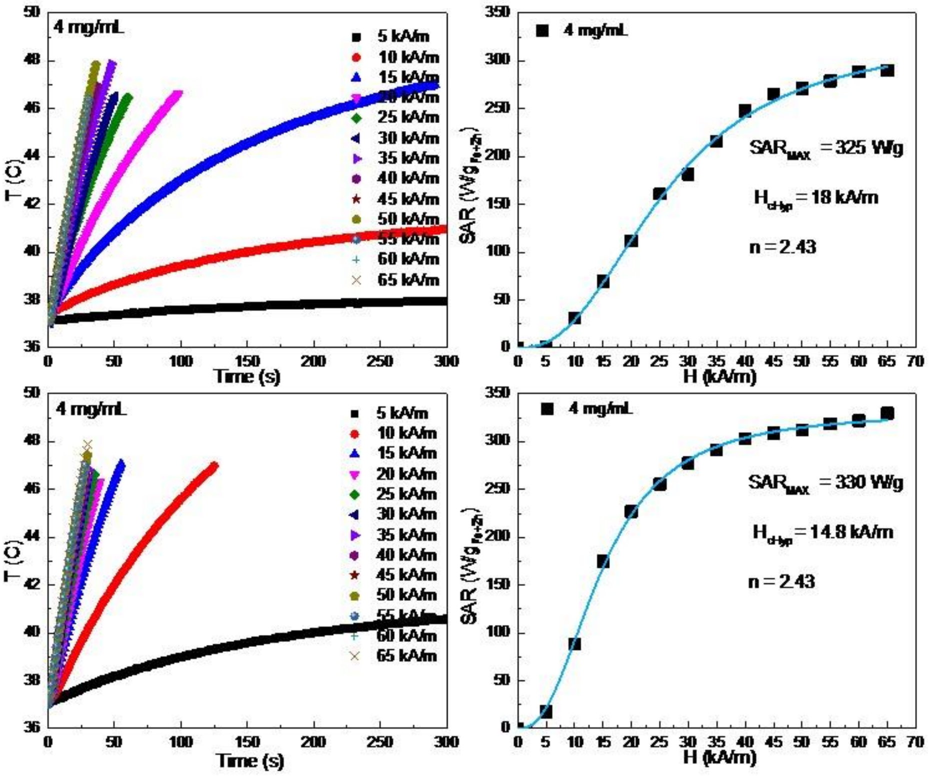

Figure S5. Left panels: Heating curves of $ZnFe_2O_4$, dispersed in PEG8K either randomly (upper panel) or pre-aligned in a static magnetic field (lower panel), recorded as a function of AC magnetic field amplitudes at 355 kHz. Right panel SAR values of $ZnFe_2O_4$ MNPs dispersed in PEG8K either randomly (upper panel) or pre-aligned in a static magnetic field (lower panel) as a function of the AMF amplitudes and at a frequency of 355 kHz. The data are fitted with a sigmoidal function (blue lines).

9. Hyperthermia experiments performed in cell culture media

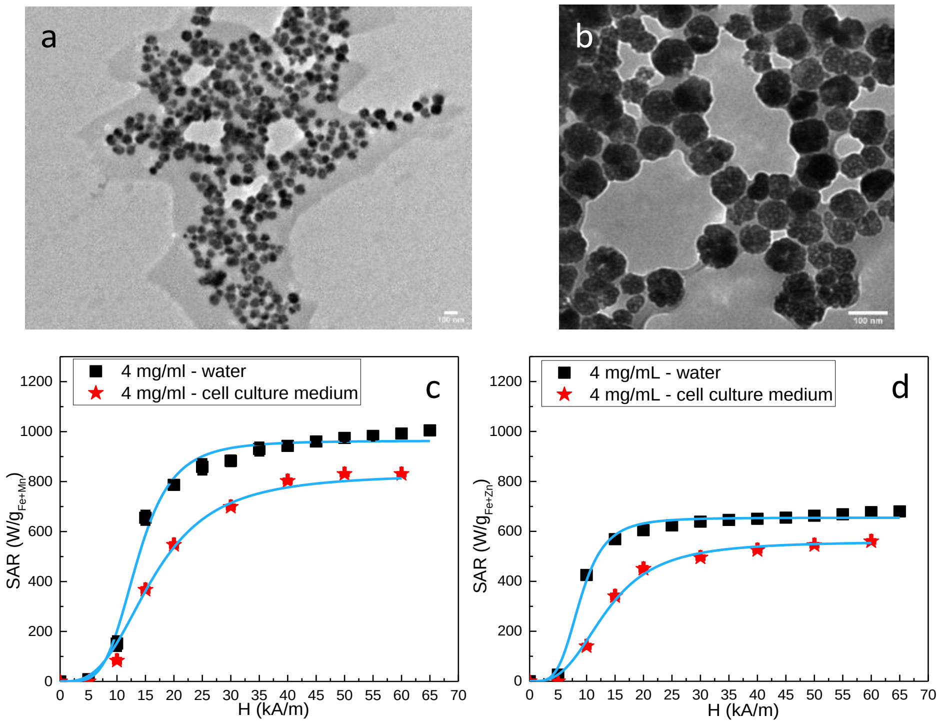

Figure S6. Large-scale (a) and zoom in (b) TEM images of $MnFe_2O_4$ MNPs dispersed in cell culture medium. SAR dependence on the alternating magnetic field amplitude for manganese ferrite (c) and zinc ferrite (d) in water and cell culture medium at a concentration of 4 mg/mL.

10. Alignment of the MNPs in DC magnetic fields.

The MNPs at the desired concentrations dispersed in water were collected at the bottom of the vial by a magnet; the water was discharged and 0.5 mL liquid PEG 8K heated at 80^{0}C was introduced. The samples were immediately sonicated for 10 minutes in an ultra-sonication bath heated at 80^{0}C. Right after the samples were placed in the middle of the distance between two 1 cm cubic Neodymium magnets separated by 7 cm. The magnetic induction measured with a Gaussmeter is almost constant in a region of around 1 cm in the center of the system (between 3 cm and 4 cm from one magnet) according to the calibration curve provided in Figure SI9. The samples were left to solidify under a 15 mT DC magnetic field.

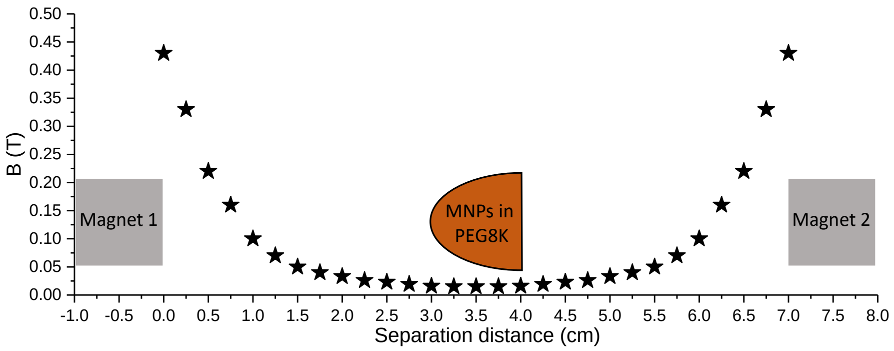

Figure S7. Magnetic induction calibration curve between two neodymium (Ne-Fe-B) magnets separated by 7 cm one from the other.

11. TEM analysis of B16F10 and A549 cells incubated with both types of MNPs

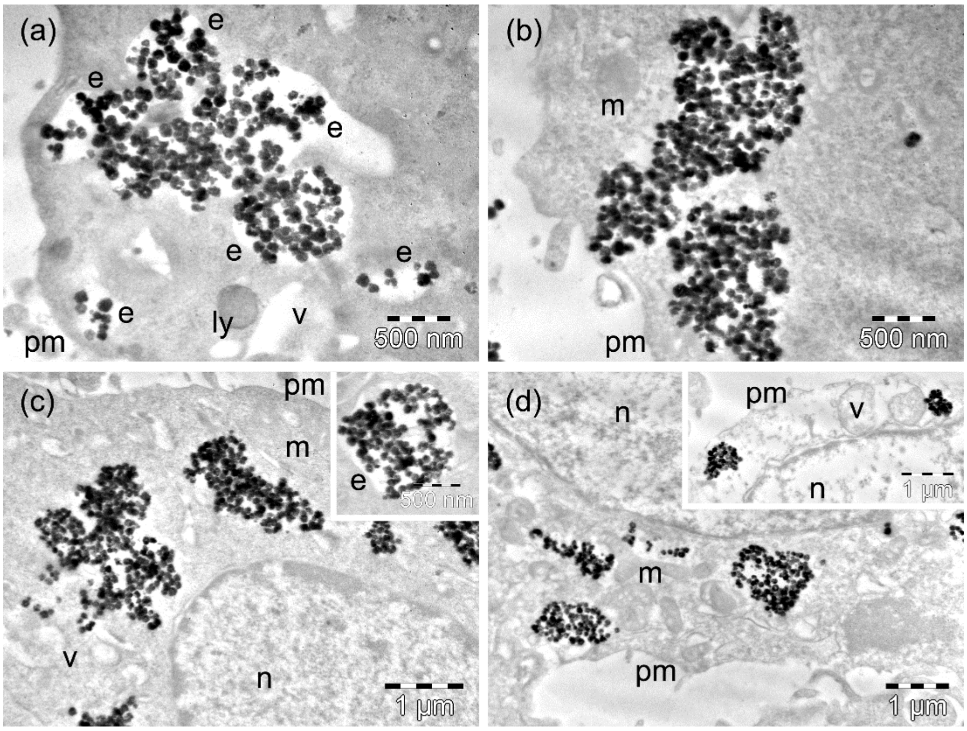

Figure S8. TEM images of B16F10 cells containing $MnFe_2O_4$ MNPs after incubation of 4h (**a** and **b**) and 24h (**c** and **d**). The letters n, e, v, ly, m, and pm denote the nucleus, the endosomes, the vacuoles, the lysosomes, mitochondria, and the plasma membranes. Insets **(c, right)** and **(d, right)** show an MNPs contained endosome and zoom in a TEM image displaying the nucleus, two MNPs contained endosomes and a vacuole, respectively. .

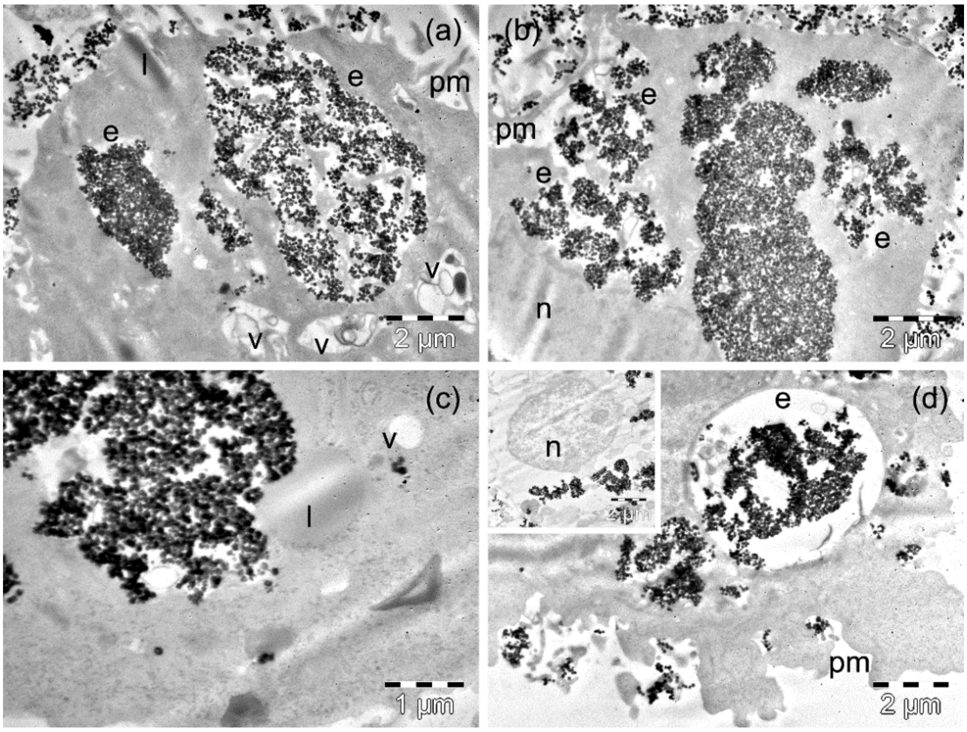

Figure S9. TEM images of A549 cells containing $MnFe_2O_4$ MNPs after incubation of 4 h (**a** and **b**) and 24 h (**c** and **d**). The letters n, e, v, ly, m, and pm denote the nucleus, the endosomes, the vacuoles, the

lysosomes, mitochondria, and the plasma membranes. Inset **(d, left)** shows a large scale TEM image displaying the nucleus and different large endosomes containing MNPs.

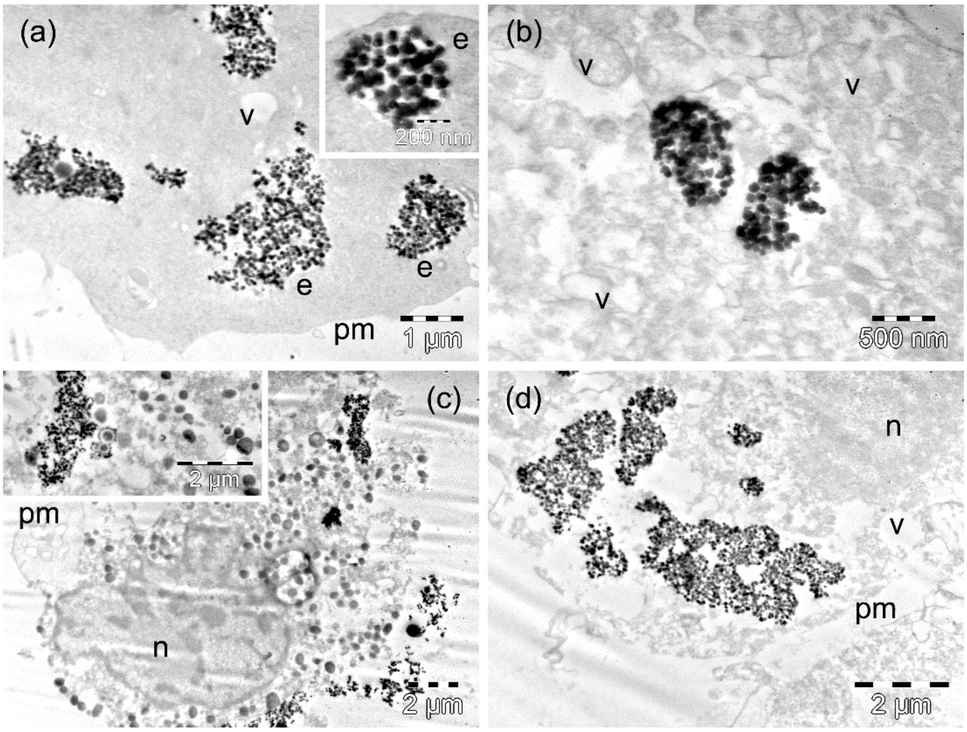

Figure S10. TEM images of B16F10 cells containing $ZnFe_2O_4$ MNPs after incubation of 4h **(a** and **b)** and 24h **(c** and **d)**. The letters n, e, v, ly, m, and pm denote the nucleus, the endosomes, the vacuoles, the lysosomes, mitochondria, and the plasma membranes. Insets **(e, right)** and **(c, left)** show an MNPs contained endosomes and other dead cells, respectively.

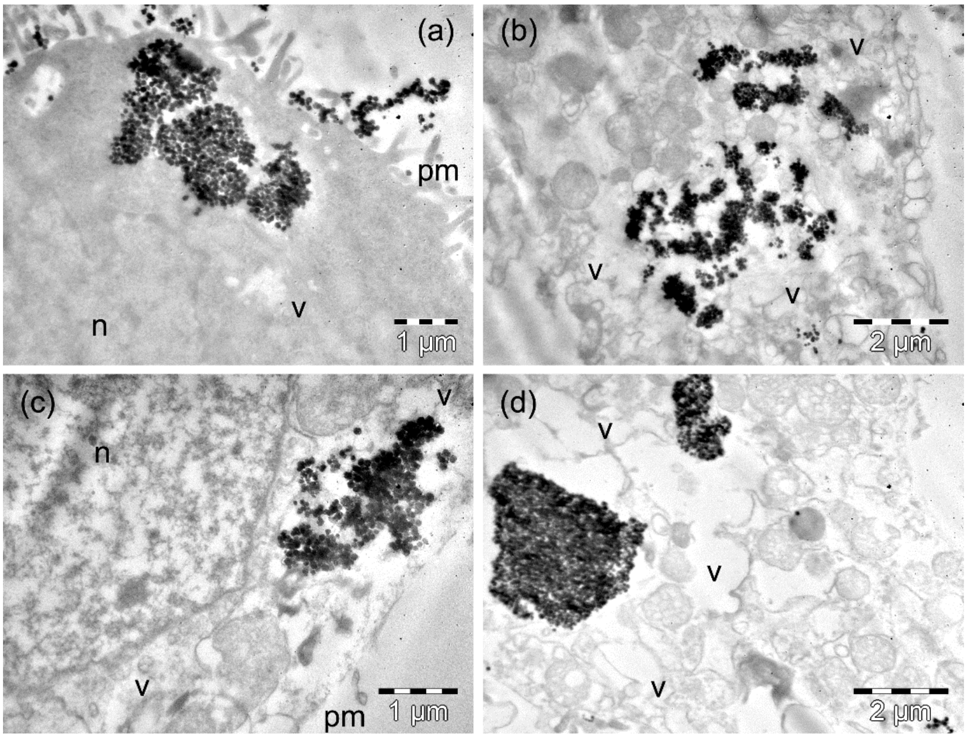

Figure S11. TEM images of A549 cells containing $ZnFe_2O_4$ MNPs after incubation of 4 h **(a** and **b)** and 24 h **(c** and **d)**. The letters n, e, v, ly, m, and pm denote the nucleus, the endosomes, the vacuoles, the lysosomes, mitochondria, and the plasma membranes.

12. Microanalysis results of the ultrastructural effects resulted from cell incubation with both types of MNPs.

Table S1. Surface area (in µm²/cell) occupied by MNPs in the four types of cells.

Cell line	$MnFe_2O_4$ MNPs				$ZnFe_2O_4$ MNPs			
	4 h		24 h		4 h		24 h	
	Mean	SE	Mean	SE	Mean	SE	Mean	SE
D407	4.35[a]	0.87	4.44	0.62	3.93	0.74	3.68	0.86
MW35	1.88[b]	0.36	1.64	0.31	1.18	0.33	0.25[g]	0.25
B16F10	4.76[c]	1.18	1.69	0.47	4.59	1.15	9.34[g]	2.45
A549	20.33[abcdef]	3.97	9.27[d]	2.6	5.02[e]	1.7	8.32[f]	1.7

Letters indicate the pairs of values between which statistical significance was calculated with ANOVA ($p<0.05$).

Table S2. Surface area (in µm²/cell) occupied by cytoplasmic vacuoles in the four types of cells.

Cell line	$MnFe_2O_4$ MNPs				$ZnFe_2O_4$ MNPs			
	4 h		24 h		4 h		24 h	
	Mean	SE	Mean	SE	Mean	SE	Mean	SE
D407	1.54	0.45	0.63	0.18	0.86	0.24	2.92[a]	0.69
MW35	0.36	0.31	6.07[b]	2.4	0.99[c]	0.34	25.23[abcde]	4.17
B16F10	0.66	0.55	0.04	0.04	2.01	0.95	4.99[d]	1.33
A549	4.43	1.12	1.14[f]	0.37	2.12	0.72	10.31[ef]	4.97

Letters indicate the pairs of values between which statistical significance was calculated with ANOVA ($p<0.05$).

References

1. Ângela L. Andrade, José D. Fabris, José D. Ardisson, Manuel A. Valente, and José M. F. Ferreira. Effect of Tetramethylammonium Hydroxide on Nucleation, Surface Modification and Growth of Magnetic Nanoparticles. *J. Nanomaterials*, **2012**, 2012, doi:10.1155/2012/454759.
